# Occupational causes of hypersensitivity pneumonitis: a systematic review and compendium

**DOI:** 10.1093/occmed/kqab082

**Published:** 2021-08-09

**Authors:** N Kongsupon, G I Walters, S S Sadhra

**Affiliations:** 1 Institute of Occupational and Environmental Medicine, College of Medical and Dental Sciences, University of Birmingham, Birmingham B152TT, UK; 2 Birmingham NHS Regional Occupational Lung Disease Service, Birmingham Chest Clinic, 151 Great Charles Street, Birmingham B3 3HX, UK

**Keywords:** Aetiology, extrinsic allergic alveolitis, occupational disease, occupational exposure, occupational health

## Abstract

**Background:**

Hypersensitivity pneumonitis (HP) is caused by a variety of antigens and low-molecular-weight chemicals, often through occupational exposure. Making a diagnosis of HP and identifying a cause are challenging. Cryptogenic cases are frequently reported, and missing or incomplete exposure histories can cause misclassification.

**Aims:**

To provide an evidence-based compendium of sources of exposure and causes of HP for the clinician, through systematic review of medical literature.

**Methods:**

Articles related to HP causative agents and occupational exposure were searched from the databases OVID Medline (1946 to October 2020) and EMBASE (1974 to October 2020). Abstracts and full texts of articles were screened by two reviewers. Data on causative antigens, occupational source of exposure and any associated eponymous name were extracted and grouped according to source of exposure.

**Results:**

A total of 1790 articles were identified, from which 305 articles met the inclusion criteria. An additional 22 articles were identified from citation lists of the selected review articles. Sources of exposure identified for HP were sorted into 14 categories of work (agricultural, plant matter processing, wood, animal-related, foodstuff, food processing, metal processing, polymers, other manufacturing, chemicals, aerosolized water, service, waste and sewage and wind instruments).

**Conclusions:**

This work is a comprehensive list of occupational causative agents and exposures causing HP. Cases are grouped by source of exposure, allowing an immediately accessible compendium of causes for use during occupational exposure assessment, which could also form the basis for a clinical questionnaire.

**Key learning points**

**What is already known about this subject:**

- Hypersensitivity pneumonitis is caused by a variety of inhaled antigens and haptens, often through occupational exposures.
- Identifying the causative antigen and a relevant source of exposure is one of the most important factors in making an accurate diagnosis of hypersensitivity pneumonitis and for prognosis.
- Cryptogenic hypersensitivity pneumonitis is often reported, and missing or incomplete exposure histories can cause misclassification of the disease.

**What this study adds:**

- This is a compendium of occupational causes and sources of exposure for hypersensitivity pneumonitis, derived by systematic review of the medical literature.

**What impact this may have on practice or policy:**

- A compendium of cases of hypersensitivity pneumonitis sorted by industry, work process or substance used may provide a reference for the practising clinician during occupational exposure assessment.
- This could form the basis for an exposure assessment questionnaire, where the cause for hypersensitivity pneumonitis is not clear.

## Introduction

Hypersensitivity pneumonitis (HP) is an inflammatory response occurring in the alveoli and bronchi following exposure to an inhaled organic antigen or low-molecular-weight agent. Pepys and Jenkins [1] first demonstrated precipitating antibodies to mouldy hay in farmers in the 1960s. Since then, many antigens and exposures have been identified as causes, from case reports, series and surveillance reports; these have given rise to labels based on occupation, such as cheese washers’ or mushroom workers’ lung. In the UK, the most frequently reported causative exposure from occupational health surveillance is now metalworking fluid, having replaced farmers’ lung over the last two decades [2].

Diagnosis of HP is based on exposure history, computerized tomographic appearance, bronchiolo-alveolar differential cell count, with or without histology, and demonstration of sensitization to a known antigen, by specific immunoglobulin G or precipitating antibodies. Diagnosis of HP can be difficult since radiological appearance is not specific to the disease, exposure to a known cause is not always apparent and specific sensitization can occur in the absence of lung disease. A recent Delphi study [3] among an international group of experts identified the most important discriminatory features in making a diagnosis; these comprised exposure to a known antigen, a suitable temporal relationship between exposure and disease, and improvement on avoidance of antigen exposure. However, ‘cryptogenic’ HP is frequently reported and exposure histories are often missing or incomplete [4–7]. In acute HP, antigen avoidance leads to better outcomes, though this is not necessarily the case for more fibrotic HP, where the natural history varies in response to antigen exposure, and the role of historical exposures is unclear [8, 9]. We aim to produce a comprehensive list of causative agents derived from occupational exposures for HP by systematic review of published medical literature.

## Methods

**Figure 1.**
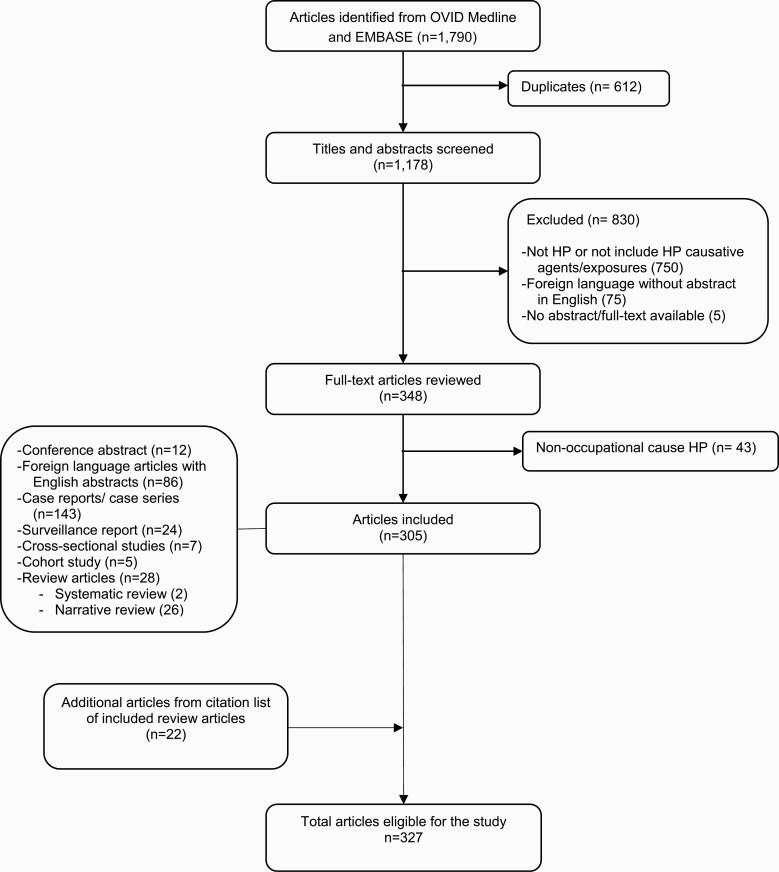
PRISMA diagram for systematic review of occupational causative agents implicated in diagnoses of HP [10].

A systematic evidence review was conducted by searching the databases OVID Medline (1946 until October 2020) and EMBASE (1974 until October 2020). A flow diagram is shown in Figure 1. The following search terms were used with no limits or filters: ‘hypersensitivity pneumonitis OR extrinsic allergic alveolitis OR allergic alveolitis) AND (occupation OR occupational OR work-related)’. Search results were imported to EndNote software and duplicates were removed and then exported to a Microsoft Excel spreadsheet for the study selection process. Two reviewers (N.K. and S.S.S.) individually screened the titles and abstracts; full-text articles were reviewed when abstracts were not present or available. Articles were eligible if they met the following criteria: (i) included individuals who were diagnosed with HP, (ii) causative exposures were occupational in origin and (iii) abstracts were written in English.

Eligible articles were categorized as conference abstracts, foreign language articles with English abstracts, case reports and series, surveillance reports, cross-sectional studies, cohort studies and review articles (narrative or systematic). The citation lists of included review articles were screened for additional causative agents and exposures. For each included article (authors, year, journal, study design), data on causative antigen or hapten, occupational source of exposure (work process, industry, substance encountered) and any given name for the disease were extracted by N.K.; antigens and haptens were grouped according to source of exposure. As a measure of quality control, included articles were categorized into peer and non-peer reviewed. ‘Peer reviewed’ was defined as: identified in ‘Web of Science Core Collection’ database (Clarivate Analytics, Philadelphia, PA, USA) or assigned as ‘refereed’ in ‘Ulrichsweb’ database (ProQuest, Ann Arbor, MI, USA). ‘Non-peer reviewed’ was defined as either not listed in the two databases above, or a conference abstract. Ethical review was not sought for this literature review, since the study did not involve NHS patients or workers.

## Results

The database search identified 1790 articles. After removing the duplicates and irrelevant articles, 305 articles were eligible for the study; 22 additional articles were included from citation lists of review articles (Figure 1). Sources of exposure were sorted into 14 categories (see Table 1, available as Supplementary data at *Occupational Medicine* Online). There were 158 different antigens or haptens, and 125 occupational sources of HP identified from the review; out of these, 17 antigens or haptens and 9 occupational sources were evidenced by non-peer-reviewed references. For 17 exposures, causative antigens or haptens remained unidentified or were not reported in the articles. Five sources were inferred from review articles as original articles were unavailable. Citations for all included articles are listed in Supplementary File (available as Supplementary data at *Occupational Medicine* Online).

## Discussion

We have undertaken a systematic review of the medical literature, which, in summary, has found 305 eligible articles and additional 22 articles from citation lists of the review articles. We have identified 158 antigens or haptens and 125 occupational sources which were grouped into 14 categories. A handful of occupational sources were derived from non-peer-reviewed articles. This is a comprehensive list of reported occupational causative agents and exposures for HP. Evidence is based largely on case series and reports, with a small number of cross-sectional or cohort studies. Causes are grouped by source of exposure (industry, work process or substances encountered) rather than a specific antigen or hapten, which is likely to be of benefit to the practising clinician searching for an identifiable cause for HP in their worker or patient. This is an arbitrary categorization, derived iteratively from examining previous reports, and not based on molecular structure or reactivity, so not intended to specifically evaluate the causal relationship.

There are several other limitations to this study. Firstly, the case definition for HP has not been pre-determined and assessed in each study. This is because HP diagnosis may be complex, due to non-specific clinical manifestations that mimic other interstitial lung diseases, variable natural history, variable radiological appearance and lack of universal diagnostic criteria. Secondly, the level of evidence is generally low with predominance of case reports and series, and there has been no individual assessment of quality of included clinical studies; rating was based upon whether an article had been peer reviewed or not.

However, the available information from many national registries shows that HP incidence is rare, making reports of individual cases and exposures important, and so a low threshold for inclusion was pre-established. In the UK general population, about nine cases per million person-years is an estimated incidence from a primary care database [11], while the number is reduced to one to two cases per million workers from the national voluntary reporting scheme by occupational and respiratory specialists [2]. In Europe, the incidence ranges from 0.35 to 12.2 cases per million workers; in high-risk occupations, HP cases could be as high as 10–13% (e.g. farmers, pigeon breeders) [12]. These numbers are in fact derived from different baseline populations (insurance-/health service-/workplace-based), reporting systems (voluntary/obligatory) and most importantly different diagnostic criteria; many HP cases are still under- or misdiagnosed.

To make an accurate diagnosis of HP and identify the cause, thorough occupational and environmental histories are essential; exposure enquiries are likely to be limited by individual prior clinical experience in the absence of a standardized assessment tool. Furthermore, in a large HP case series derived from interstitial lung disease multidisciplinary team discussion and consensus, exposure histories of patients with cryptogenic HP were missing in 20–30% [4]. Morell *et al*. [13] reported that 40% of patients who had a primary diagnosis of idiopathic pulmonary fibrosis were subsequently diagnosed with HP, following identification of occult exposures to feather bedding. Therefore, there is a good argument for further research to validate and examine the effectiveness of a systematically derived exposure questionnaire.

The difficulty here is, as this compendium demonstrates, that the number of causes and exposures is large and various, and would make a questionnaire cumbersome. Barnes *et al*. [14] have identified a small number of causative exposures (18) suitable for screening questionnaire, by systematic review of medical literature (exposures were included if reported in five or more cases), then studied by Delphi to determine their importance among experts. However, the sensitivity and specificity of this tool have not been established. As a rare disease with many and various causes, a compendium is a useful tool, particularly in the setting of occupational HP, where an occupational cause is likely if working-age patients present with work-related symptoms of acute or sub-acute duration [4].

## Funding

There was no funding for this study.

## Competing interests

None declared.

## Supplementary Material

kqab082_suppl_Supplementary_File
