# Supplementary material for: Occupational causes of hypersensitivity pneumonitis: a systematic review and compendium
Source: Occup Med (Lond). 2021 Aug 9;71(6-7):255–9. doi: 10.1093/occmed/kqab082 (PMC8486273; doi:10.1093/occmed/kqab082)
Supplement: kqab082_suppl_Supplementary_File [file kqab082_suppl_supplementary_file.docx]

**Supplementary file**

**Occupational causes of hypersensitivity pneumonitis: a compendium**

Ngamjit Kongsupon^1^, Gareth I. Walters^1,2^, Steven Sadhra^1^

^1^ Institute of Occupational and Environmental Medicine, College of Medical and Dental Sciences, University of Birmingham, Birmingham, UK, B152TT

^2^ Birmingham NHS Regional Occupational Lung Disease Service, Birmingham Chest Clinic, 151 Great Charles Street, Birmingham, UK, B3 3HX

**Citations for articles included in Table 1**

ABREU J.P., ESTEVES J., BONCORAGLIO M.T., PEREIRA F.M., COSTA C., OLIVEIRA C. 2020. Cladosporium herbarum hot-tub lung hypersensitivity pneumonitis in a greenhouse worker. *European Journal of Case Reports in Internal Medicine.* 7, (no pagination).

AKIMOTO, T., TAMURA, N., UCHIDA, K., DAMBARA, T., NUKIWA, T. & KIRA, S. 1992. [A case of hypersensitivity pneumonitis due to isocyanate exposure showing progression even two months after removal of the antigen]. *Nihon Kyobu Shikkan Gakkai zasshi,* 30**,** 458-63.

AKIZUKI, N., INASE, N., ISHIWATA, N., JIN, Y., ATARASHI, K., ICHIOKA, M., YOSHIZAWA, Y. & MARUMO, F. 1999. Hypersensitivity pneumonitis among workers cultivating Tricholoma conglobatum (shimeji). *Respiration,* 66**,** 273-8.

AMANO, Y., ENOMOTO, M., BANDO, M., KAWAKAMI, M. & SUGIYAMA, Y. 2009. [Hypersensitity pneumonitis in a greenhouse rose grower]. *Nihon Kokyuki Gakkai zasshi,* 47**,** 960-4.

AMEILLE, J., BRECHOT, J. M., BROCHARD, P., CAPRON, F. & DORE, M. F. 1992. Occupational hypersensitivity pneumonitis in a smelter exposed to zinc fumes. *Chest,* 101**,** 862-863.

AMPERE, A., DELHAES, L., SOOTS, J., BART, F. & WALLAERT, B. 2012. Hypersensitivity pneumonitis induced by Shiitake mushroom spores. *Medical Mycology,* 50**,** 654-7.

ANDO, M., YOSHIDA, K., NAKASHIMA, H., SUGIHARA, Y. & KASHIDA, Y. 1994. Role of Candida albicans in Chronic Hypersensitivity Pneumonitis. *Chest,* 105**,** 317-318.

ARNOW, P. M., FINK, J. N., SCHLUETER, D. P., BARBORIAK, J. J., MALLISON, G., SAID, S. I., MARTIN, S., UNGER, G. F., SCANLON, G. T. & KURUP, V. P. 1978. Early detection of hypersensitivity pneumonitis in office workers. *American Journal of Medicine,* 64**,** 236-42.

ÁVILA, R. 1971. Extrinsic allergic alveolitis in workers exposed to fish meal and poultry. *Clinical & Experimental Allergy,* 1**,** 343-346.

AYDEMIR, Y., GUNGEN, A. C. & COBAN, H. 2015. Hypersensitivity pneumonitis caused by the broom grass (Callunavulgaris). *Respiratory Medicine Case Reports,* 15**,** 135-137.

BANDO, T., NODA, Y., HIROSE, J., OHTA, G., SHIBATA, K., FUJIMURA, M. & MATSUDA, T. 1993. [A case of hypersensitivity pneumonitis induced by toluene diisocyanate presenting with transient bronchoconstriction]. *Nihon Kyobu Shikkan Gakkai zasshi,* 31**,** 1297-302.

BARRANCO, P., MORENO-ANCILLO, A., ROBLES, M. L. M., ALVAREZ-SALA, R., DE TERRERAS, L. G., MARTIN-ESTEBAN, M., VICENTE, J. & LOPEZ-SERRANO, M. C. 1999. Hypersensitivity pneumonitis in a worker exposed to tiger nut dust. *Journal of Allergy and Clinical Immunology,* 104**,** 500-501.

BARRETT, M. & HAYES, J. 2012. Bird fancier's lung in mushroom workers. *Irish Journal of Medical Science,* 181**,** S388.

BARTIZALOVA, S. 2012. Extrinsic allergic alveolitis. *Interni Medicina pro Praxi,* 14**,** 383-386.

BARZO, P., MOLNAR, L. & CSOKONAY, L. 1989. [Allergic alveolitis in agricultural workers, caused by thermophilic bacteria or fungi]. *Zeitschrift fur Erkrankungen der Atmungsorgane,* 173**,** 151-160.

BAUR, X. 1995. Hypersensitivity pneumonitis (extrinsic allergic alveolitis) induced by isocyanates. *Journal of Allergy and Clinical Immunology,* 95**,** 1004-1010.

BAUR, X., CHEN, Z. & MARCZYNSKI, B. 2001. Respiratory diseases caused by occupational exposure to 1,5-naphthalene-diisocyanate (NDI): Results of workplace-related challenge tests and antibody analyses. *American Journal of Industrial Medicine,* 39**,** 369-372.

BAUR, X., DEWAIR, M. & ROMMELT, H. 1984. Acute airway obstruction followed by hypersensitivity pneumonitis in an isocyanate (MDI) worker. *Journal of Occupational Medicine,* 26**,** 285-287.

BAUR, X., GAHNZ, G. & CHEN, Z. 2000. Extrinsic allergic alveolitis caused by cabreuva wood dust. *Journal of Allergy and Clinical Immunology,* 106**,** 780-781.

BECKETT, W., KALLAY, M., SOOD, A., ZUO, Z. & MILTON, D. 2005. Hypersensitivity pneumonitis associated with environmental mycobacteria. *Environmental Health Perspectives,* 113**,** 767-770.

BELLANGER, A. P., MORISSE-PRADIER, H., REBOUX, G., SCHERER, E., PRAMIL, S., DOMINIQUE, S. & MILLON, L. 2019. Hypersensitivity pneumonitis in a cystic fibrosis patient. *Occupational Medicine,* 69**,** 632-634.

BENZARTI MEZNI, A., MHIRI, N., BEJI, M. & BEN JEMAA, A. 2010. [Hypersensitivity pneumonitis due to proteolytic enzymes of Bacillus subtilis : A case report]. *Revue Francaise d'Allergologie,* 50**,** 77-81.

BERGH, K. 1982. [Hypersensitivity pneumonitis due to diphenylmethane diisocyanate (MDI) exposure. A case report]. *Tidsskrift for den Norske Legeforening,* 102**,** 380.

BERGMANN CH, K. 1979. [Organisation and results from the Center for Exogenous Allergic Alveolitis in the GDR]. *Atemwegs- und Lungenkrankheiten,* 5**,** 379-381.

BERNSTEIN, D. I., LUMMUS, Z. L., SANTILLI, G., SISKOSKY, J. & BERNSTEIN, I. L. 1995. Machine operator's lung: A hypersensitivity pneumonitis disorder associated with exposure to metalworking fluid aerosols. *Chest,* 108**,** 636-641.

BERNSTEIN, R. S., SORENSON, W. G., GARABRANT, D., REAUX, C. & TREITMAN, R. D. 1983. Exposures to respirable, airborne Penicillium from a contaminated ventilation system: Clinical, environmental and epidemiological aspects. *American Industrial Hygiene Association Journal,* 44**,** 161-169.

BIELER, G., THORN, D., HUYNH, C. K., TOMICIC, C., STEINER, U. C., YAWALKAR, N. & DANUSER, B. 2011. Acute life-threatening extrinsic allergic alveolitis in a paint controller. *Occupational Medicine,* 61**,** 440-442.

BOCCHIA, M. E., CAPATO, S., PESSINA, I., SPAGNOTTO, S. & VAGHI, A. 2005. [Hypersensitivity pneumonitis in budgerigar fanciers. A seldom recognised disease?]. *Recenti Progressi in Medicina,* 96**,** 293-4.

BOYADZIEVA, V. I., STOILOV, N., KUNEVA, T. & STOILOV, R. 2014. A clinical case of chronic hypersensitivity pneumonitis due to occupational exposure to organic dust (coffee). *Revmatologiia (Bulgaria),* 22**,** 42-48.

BRACKER, A., STOREY, E., YANG, C. & HODGSON, M. J. 2003. An outbreak of hypersensitivity pneumonitis at a metalworking plant: A longitudinal assessment of intervention effectiveness. *Applied Occupational and Environmental Hygiene,* 18**,** 96-108.

BRINGGOLD, W., HALLIDAY, S. 2020. The cheese whiz lung: hypersensitivity pneumonitis in an imitation cheese factory worker. *Chest.* 158, A1821.

BROOKS, J., HESS, M., BENZ, J. & MACDONALD, J. 2017. Hypersensitivity pneumonitis in a farmer. *World Allergy Organization Journal,* 10**,** 25.

BRUN, J., BRIAND, P. & GUILLAIS, P. 1979. Two cases of extrinsic allergic alveolitis. *Ouest Medical,* 32**,** 551-558.

BUICK, J. B. & TODD, G. R. G. 1997. Concomitant alveolitis and asthma following exposure to triphenylmethane triisocyanate. *Occupational Medicine,* 47**,** 504-506.

BUNGER, J., SCHAPPLER-SCHEELE, B., HILGERS, R. & HALLIER, E. 2007. A 5-year follow-up study on respiratory disorders and lung function in workers exposed to organic dust from composting plants. *International Archives of Occupational and Environmental Health,* 80**,** 306-312.

CAMPBELL, J. A., KRYDA, M. J., TREUHAFT, M. W., MARX, J. J., JR. & ROBERTS, R. C. 1983. Cheese worker's hypersensitivity pneumonitis. *American Review of Respiratory Disease,* 127**,** 495-6.

CAMPBELL, J. M. 1932. Acute Symptoms Following Work With Hay. *British Medical Journal,* 2**,** 1143-1144.

CARROLL, K. B., PEPYS, J., LONGBOTTOM, J. L., HUGHES, D. T. & BENSON, H. G. 1975. Extrinsic allergic alveolitis due to rat serum proteins. *Clinical allergy,* 5**,** 443-56.

CATENACCI, G., LODIGIANI, L. & TRINGALI, S. 1990. [Extrinsic allergic alveolitis of occupational origin: apropos of 2 cases]. *Giornale Italiano di Medicina del Lavoro,* 12**,** 37-42.

CENTERS FOR DISEASE CONTROL AND PREVENTION 1996. Biopsy-confirmed hypersensitivity pneumonitis in automobile production workers exposed to metalworking fluids--Michigan, 1994-1995. *Morbidity and Mortality Weekly Report,* 45**,** 606-610.

CENTERS FOR DISEASE CONTROL AND PREVENTION 2002. Respiratory illness in workers exposed to metalworking fluid contaminated with nontuberculous mycobacteria--Ohio, 2001. *Morbidity and Mortality Weekly Report,* 51**,** 349-52.

CHANNELL, S., BLYTH, W., LLOYD, M., WEIR, D. M., AMOS, W. M., LITTLEWOOD, A. P., RIDDLE, H. F. & GRANT, I. W. 1969. Allergic alveolitis in maltworkers. A clinical, mycological, and immunological study. *Quarterly Journal of Medicine,* 38**,** 351-76.

CHASSE, M., BLANCHETTE, G., MALO, J. & MALO, J. L. 1986. Farmer's lung presenting as respiratory failure and homogeneous consolidation. *Chest,* 90**,** 783-784.

CHIBA, S., OKADA, S., SUZUKI, Y., WATANUKI, Z., MITSUISHI, Y., IGUSA, R., SEKII, T. & UCHIYAMA, B. 2009. *Cladosporium* Species-Related Hypersensitivity Pneumonitis in Household Environments. *Internal Medicine,* 48**,** 363-367.

CHOY, A. C., PATTERSON, R., RAY, A. H. & ROBERTS, M. 1995. Hypersensitivity pneumonitis in a raptor handler and a wild bird fancier. *Annals of Allergy, Asthma and Immunology,* 74**,** 437-441.

CLOETE, B. 2014. Isocyanate exposure - An unusual case of spray painter's lung. *Current Allergy and Clinical Immunology,* 27**,** 322-327.

COHEN, H. I., MERIGAN, T. C., KOSEK, J. C. & ELDRIDGE, F. 1967. Sequoiosis: A granulomatous pneumonitis associated with redwood sawdust inhalation. *American Journal of Medicine,* 43**,** 785-794.

COLIN, G., LELONG, J., TILLIE-LEBLOND, I. & TONNEL, A. B. 2007. [Hypersensitivity pneumonitis in a chicory worker]. *Revue des Maladies Respiratoires,* 24**,** 1139-42.

CORMIER, Y., ISRAEL-ASSAYAG, E., BEDARD, G. & DUCHAINE, C. 1998. Hypersensitivity pneumonitis in peat moss processing plant workers. *American Journal of Respiratory and Critical Care Medicine,* 158**,** 412-417.

CRUZ, M. J., MORELL, F., ROGER, A., MUNOZ, X. & RODRIGO, M. J. 2003. [Hypersensitivity pneumonitis in construction plasterers (espartosis): Study of 20 patients]. *Medicina Clinica,* 120**,** 578-583.

D'SOUZA, R. S. & DONATO, A. 2017. Hypersensitivity pneumonitis: an overlooked cause of cough and dyspnea. *Journal of Community Hospital Internal Medicine Perspectives,* 7**,** 95-99.

DAWKINS, P., ROBERTSON, A., ROBERTSON, W., MOORE, V., REYNOLDS, J., LANGMAN, G., ROBINSON, E., HARRIS-ROBERTS, J., CROOK, B. & BURGE, S. 2006. An outbreak of extrinsic alveolitis at a car engine plant. *Occupational Medicine,* 56**,** 559-565.

DE BEUKELAAR, T., SLABBYNCK, H., UYTTENBROECK, W., VERBRUGGEN, A., DE SURGELOOSE, D. & VAN GOETHEM, J. 2015. A 36-year-old man with acute respiratory failure: A case report. *Tijdschrift voor Geneeskunde,* 71**,** 1131-1137.

DE HOYOS, A., HOLNESS, D. L. & TARLO, S. M. 1993. Hypersensitivity pneumonitis and airways hyperreactivity induced by occupational exposure to penicillin. *Chest,* 103**,** 303-304.

DEBELJAK, A. & SORLI, J. 1975. Farmer's lung. *Zdravstveni Vestnik,* 44**,** 103-106.

DESCHENES, D., PROVENCHER, S. & CORMIER, Y. 2012. Farmer's lung-induced hypersensitivity pneumonitis complicated by shock. *Respiratory Care,* 57**,** 464-466.

DIETEMANN-MOLARD, A., BRAUN, J. J., SOHIER, B. & PAULI, G. 1991. Extrinsic allergic alveolitis secondary to carmine. *Lancet,* 338**,** 460.

DUTKIEWICZ, J., KUS, L., DUTKIEWICZ, E. & WARREN, C. P. 1985. Hypersensitivity pneumonitis in grain farmers due to sensitization to Erwinia herbicola. *Annals of Allergy,* 54**,** 65-8.

DYKEWICZ, M. S., LAUFER, P., PATTERSON, R., ROBERTS, M. & SOMMERS, H. M. 1988. Woodman's disease: Hypersensitivity pneumonitis from cutting live trees. *Journal of Allergy and Clinical Immunology,* 81**,** 455-460.

EBNER, H., FELDNER, H. & KRAFT, D. 1981. [Some observations on farmer's lung]. *Wiener Klinische Wochenschrift,* 93**,** 729-733.

ELLIS, M. E. & FRIEND, J. A. 1981. Progressive lung disease in a malt-worker. *Thorax,* 36**,** 552-3.

EVANS, W. V. & SEATON, A. 1979. Hypersensitivity pneumonitis in a technician using Pauli's reagent. *Thorax,* 34**,** 767-70.

FAERDEN, K., BRIT LUND, M., MOGENS AALOKKEN, T., EDUARD, W., SOSTRAND, P., LANGARD, S. & KONGERUD, J. 2014. Hypersensitivity pneumonitis in a cluster of sawmill workers: a 10-year follow-up of exposure, symptoms, and lung function. *International Journal of Occupational and Environmental Health,* 20**,** 167-173.

FENCLOVA, Z., PELCLOVA, D., URBAN, P., NAVRATIL, T., KLUSACKOVA, P. & LEBEDOVA, J. 2009. Occupational hypersensitivity pneumonitis reported to the Czech national registry of occupational diseases in the period 1992-2005. *Industrial Health,* 47**,** 443-448.

FERRI, F., RUGGIERI, M. P., GUIDETTI, G., AZZARONE, G., GIAMMARTINI, P., CAPANNI, S., MANTOVANI, P. & BERTANI, M. 2003. [Prevalence of allergic extrinsic alveolitis in cattle breeders in Reggio Emilia province]. *Medicina del Lavoro,* 94**,** 380-390.

FINK, J. N. & SCHLUETER, D. P. 1978. Bathtub refinisher's lung: An unusual response to toluene diisocyanate. *American Review of Respiratory Disease,* 118**,** 955-959.

FINK, J. N., SOSMAN, A. J., BARBORIAK, J. J., SCHLUETER, D. P. & HOLMES, R. A. 1968. Pigeon breeders' disease. A clinical study of a hypersensitivity pneumonitis. *Annals of Internal Medicine,* 68**,** 1205-19.

FLANDES, J., HEILI, S., SECO, J. G., SABILLON, O., FERNANDEZ, I. & ORTEGA, A. 2004. Hypersensitivity pneumonitis caused by esparto dust in a young plaster worker: A case report and review of the literature. *Respiration,* 71**,** 421-423.

FORST, L. S. & ABRAHAM, J. 1993. Hypersensitivity pneumonitis presenting as sarcoidosis. *British Journal of Industrial Medicine,* 50**,** 497-500.

FOX, J., ANDERSON, H., MOEN, T., GRUETZMACHER, G., HANRAHAN, L. & FINK, J. 1999. Metal working fluid-associated hypersensitivity pneumonitis: An outbreak investigation and case-control study. *American Journal of Industrial Medicine,* 35**,** 58-67.

FRANK, A. 1982. [Exogenous allergic alveolitis of farmers (farmer's lung) from the viewpoint of an alpine pulmologic practice]. *Wiener Medizinische Wochenschrift,* 70**,** 1-33.

FREEMAN, A., LOCKEY, J., HAWLEY, P., BIDDINGER, P. & TROUT, D. 1998. Hypersensitivity pneumonitis in a machinist. *American Journal of Industrial Medicine,* 34**,** 387-392.

FRIEND, J. A., GADDIE, J., PALMER, K. N., PICKERING, C. A. & PEPYS, J. 1977. Extrinsic allergic alveolitis and contaminated cooling-water in a factory machine. *Lancet,* 1**,** 297-300.

FUJIE, T., INASE, N., YAMADA, T., MIYASHITA, Y., OHTANI, Y. & YOSHIZAWA, Y. 2004. A case of acute bird fancier's lung (acute feather duvet lung) caused by manufacturing feather duvets. *Japanese Journal of Chest Diseases,* 63**,** 194-199.

FUJIWARA, K., SATO, T., YONEI, T., GENBA, K., NOGAMI, N. & YAMADORI, I. 2000. [A case of chronic hypersensitivity pneumonitis induced by shiitake mushroom spores]. *Nihon Kokyuki Gakkai zasshi,* 38**,** 908-913.

GALA ORTIZ, G., MENDEZ LOPEZ, J. A. & FERNANDEZ ORDONEZ, R. 1999. Hypersensitivity pneumonitis in a butcher. *Alergologia e Inmunologia Clinica,* 14**,** 330-334.

GALLAND, C., REYNAUD, C., DE HALLER, R., POLLA, B. S. & LEUENBERGER, P. 1991. [Cheese-washer's disease. A current stable form of extrinsic allergic alveolitis in a rural setting]. *Revue des Maladies Respiratoires,* 8**,** 381-6.

GAMBOA, P. M., DE LAS MARINAS, M. D., ANTEPARA, I., JAUREGUI, I. & SANZ, M. M. 1990. Extrinsic allergic alveolitis caused by esparto (Stipa tenacissima). *Allergologia et Immunopathologia,* 18**,** 331-334.

GANIER, M., LIEBERMAN, P., FINK, J. & LOCKWOOD, D. G. 1980. Humidifier lung. An outbreak in office workers. *Chest,* 77**,** 183-7.

GERBER, P., DE HALLER, R., PYROZYNSKI, W. J., STURZENEGGER, E. R. & BRANDLI, O. 1981. [Humidifier lung]. *Schweizerische medizinische Wochenschrift,* 111**,** 182-6.

GERFAUD-VALENTIN, M., REBOUX, G., TRACLET, J., THIVOLET-BEJUI, F., CORDIER, J.-F. & COTTIN, V. 2014. Occupational hypersensitivity pneumonitis in a baker: a new cause. *Chest,* 145**,** 856-858.

GERMANAUD, J., PROFFIT, V., JANVOIE, B., LEMARIE, E. & LASFARGUES, G. 2003. [Pneumopathy due to isocyanate hypersensitivity: Recognition as an occupational disease]. *Revue des Maladies Respiratoires,* 20**,** 443-449.

GERNHOLD, M. & SENNEKAMP, J. 2010. [Extrinsic allergic alveolitis caused by baker's yeast (Saccharomyces cerevisiae)]. *Allergologie,* 33**,** 579-580.

GRANT, I. W., BLACKADDER, E. S., GREENBERG, M. & BLYTH, W. 1976. Extrinsic allergic alveolitis in Scottish maltworkers. *British Medical Journal,* 1**,** 490-3.

GREENE, J. J. & BANNAN, L. T. 1985. Potato riddler's lung. *Irish Medical Journal,* 78**,** 282-284.

GUELAND, C., FRUIT, J., VANNIMENUS, C., WALLAERT, B. & TONNEL, A. B. 1993. [Occupational extrinsic allergic alveolitis due to Aspergillus oryzae]. *Revue des Maladies Respiratoires,* 10**,** 362-5.

GUILLOT, M., BERTOLETTI, L., DEYGAS, N., RABERIN, H., FAURE, O. & VERGNON, J. M. 2008. [Dry sausage mould hypersensitivity pneumonitis: three cases]. *Revue des Maladies Respiratoires,* 25**,** 596-600.

GUMUNDSSON, G., TOMASSON, K., RAFNSSON, V., SIGFUSSON, A., ODDSSON, O. H., BJOERNSDOTTIR, U. S., KRISTJANSSON, V., HALLDORSSON, S. & HARALDSSON, H. 2002. [Diagnosing occupational diseases. Examples from shellfish industry]. *Laeknabladid,* 88**,** 909-12.

GUPTA, A. & ROSENMAN, K. D. 2006. Hypersensitivity pneumonitis due to metal working fluids: Sporadic or under reported? *American Journal of Industrial Medicine,* 49**,** 423-433.

HAAHTELA, T., RIIHIMAKI, M. & MONKARE, S. 1979. [Allergic alveolitis caused by mouldy wood dust]. *Duodecim,* 95**,** 851-854.

HAGEMEYER, O., BUNGER, J., VAN KAMPEN, V., RAULF-HEIMSOTH, M., DRATH, C., MERGET, R., BRUNING, T. & BRODING, H. C. 2013. Occupational allergic respiratory diseases in garbage workers: Relevance of molds and actinomycetes. *Advances in Experimental Medicine and Biology,* 788**,** 313-320.

HALPIN, D. M. G., GRANEEK, B. J., TURNER-WARWICK, M. & TAYLOR, A. J. N. 1994. Extrinsic allergic alveolitis and asthma in a sawmill worker: Case report and review of the literature. *Occupational and Environmental Medicine,* 51**,** 160-164.

HAMAGUCHI, R., SAITO, H., KEGASAWA, K., NAKAGAWA, A., RYUJIN, Y., NOGUCHI, S., SUGIMOTO, H., KOBAYASHI, A., YAMAZAKI, K., JIN, Y., YOSHIMURA, N., TSURIKISAWA, N. & AKIYAMA, K. 2009. [A case of hypersensitivity pneumonitis resulting from inhalation of Aspergillus niger in a greenhouse worker who raised roses]. *Nihon Kokyuki Gakkai zasshi,* 47**,** 205-11.

HANAK, V., GOLBIN, J. M. & RYU, J. H. 2007. Causes and presenting features in 85 consecutive patients with hypersensitivity pneumonitis. *Mayo Clinic Proceedings,* 82**,** 812-816.

HARA, S., YAMAMOTO, K., YODA, A., NAKAYAMA, S., SAKAMOTO, N., ISHIMATSU, Y., MUKAE, H. & KOHNO, S. 2009. [Three cases of isocyanate-induced hypersensitivity pneumonitis with different HRCT findings]. *Nihon Kokyuki Gakkai zasshi,* 47**,** 839-43.

HASHIZUME, T., NUMATA, H. & MATSUSHITA, K. 2001. [A case of pneumonitis possibly due to isocyanate associated with high levels of serum KL-6]. *Nihon Kokyuki Gakkai zasshi,* 39**,** 442-5.

HELBECQUE, Y., DIANCOURT, J. M., LEFEBVRE, Y., RIVES-LANGE, L. & VAN DER BIEST, F. 1991. About one case of extrinsic allergic alveolitis related to Trichothecium in a fur coat manufacturer. *Archives des Maladies Professionnelles de Medecine du Travail et de Securite Sociale,* 52**,** 355-358.

HENDERSON, A. K., RANGER, A. F., LLOYD, J., MCSHARRY, C., MILLS, R. J. & MORAN, F. 1984. Pulmonary Hypersensitivity in the Alginate Industry. *Scottish Medical Journal,* 29**,** 90-95.

HINES, S. E., BARKER, E. A., ROBINSON, M., KNIGHT, V., GAITENS, J., SILLS, M., DUVALL, K. & ROSE, C. S. 2015. Cross-Sectional Study of Respiratory Symptoms, Spirometry, and Immunologic Sensitivity in Epoxy Resin Workers. *Clinical and Translational Science,* 8**,** 722-728.

HINOJOSA, M., FRAJ, J., DE LA HOZ, B., ALCAZAR, R. & SUEIRO, A. 1996. Hypersensitivity pneumonitis in workers exposed to esparto grass (Stipa tenacissima) fibers. *Journal of Allergy and Clinical Immunology,* 98**,** 985-91.

HODGSON, M. J., BRACKER, A., YANG, C., STOREY, E., JARVIS, B. J., MILTON, D., LUMMUS, Z., BERNSTEIN, D. & COLE, S. 2001. Hypersensitivity pneumonitis in a metal-working environment. *American Journal of Industrial Medicine,* 39**,** 616-28.

HODGSON, M. J., MOREY, P. R., SIMON, J. S., WATERS, T. D. & FINK, J. N. 1987. An outbreak of recurrent acute and chronic hypersensitivity pneumonitis in office workers. *American Journal of Epidemiology,* 125**,** 631-8.

HOLDEN, V. K., KLIGERMAN, S. J., HASTINGS, T. G. & HINES, S. E. 2017. Unabated occupational risk in a patient with rheumatoid pulmonary fibrosis. *Occupational Medicine,* 67**,** 311-313.

HOWIE, A. D., BOYD, G. & MORAN, F. 1976. Pulmonary hypersensitivity to Ramin (Gonystylus bancanus). *Thorax,* 31**,** 585-7.

HOY, R. F., PRETTO, J. J., VAN GELDEREN, D. & MCDONALD, C. F. 2007. Mushroom worker's lung: organic dust exposure in the spawning shed. *Medical journal of Australia,* 186**,** 472-4.

HUUSKONEN, M. S., HUSMAN, K., JARVISALO, J., KORHONEN, O., KOTIMAA, M., KUUSELA, T., NORDMAN, H., ZITTING, A. & MANTYJARVI, R. 1984. Extrinsic allergic alveolitis in the tobacco industry. *British Journal of Industrial Medicine,* 41**,** 77-83.

IDEZUKA, J., IKARASHI, H., NOZAWA, S., MARUYAMA, M., SASAGAWA, M. & SUZUKI, E. 1991. [A case of isocyanate-induced hypersensitivity pneumonitis and a compression-air mask thought to be effective in its prevention]. *Arerugi,* 40**,** 704-10.

INAGE, M., TAKAHASHI, H., NAKAMURA, H., MASAKANE, I. & TOMOIKE, H. 1996. Hypersensitivity pneumonitis induced by spores of Pholiota nameko. *Internal Medicine,* 35**,** 301-4.

INASE, N., SAKASHITA, H., OHTANI, Y., SOGOU, Y., SUMI, Y., UMINO, T., USUI, Y. & YOSHIZAWA, Y. 2004. Chronic bird fancier's lung presenting with acute exacerbation due to use of a feather duvet. *Internal Medicine,* 43**,** 835-837.

ISHIGURO, T., KAWAI, S., KOJIMA, A., SHIMIZU, Y., KAMEI, K. & TAKAYANAGI, N. 2018. Occupational hypersensitivity pneumonitis in a koji brewer. *Clinical Case Reports,* 6**,** 461-464.

ISHIGURO, T., TAKAYANAGI, N., YONEDA, K., MIYAHARA, Y., YANAGISAWA, T. & SUGITA, Y. 2010. [Case of bird-related hypersensitivity pneumonitis in a farmer]. *Nihon Kokyuki Gakkai zasshi,* 48**,** 985-989.

ISHIGURO, T., YASUI, M., NAKADE, Y., KIMURA, H., KATAYAMA, N., KASAHARA, K. & FUJIMURA, M. 2007. Extrinsic allergic alveolitis with eosinophil infiltration induced by 1,1,1,2-tetrafluoroethane (HFC-134a): A case report. *Internal Medicine,* 46**,** 1455-1457.

ISHII, M., KIKUCHI, A., KUDOH, K., KONISHI, K., MOHRI, T., TAMURA, M. & TOMICHI, N. 1994. Hypersensitivity pneumonitis induced by inhalation of mushroom (Pholiota nameko) spores. *Internal Medicine,* 33**,** 683-5.

JAGER, J., LIEBETRAU, G., PIELESCH, W., BERGMANN, L. & BAUR, X. 1991. [Pearl oyster shell dust as a cause of allergic alveolitis]. *Pneumologie,* 45**,** 804-806.

JAMES, P. L., CANNON, J., BARBER, C. M., CRAWFORD, L., HUGHES, H., JONES, M., SZRAM, J., COWMAN, S., COOKSON, W. O. C., MOFFATT, M. F. & CULLINAN, P. 2018. Metal worker's lung: Spatial association with Mycobacterium avium. *Thorax,* 73**,** 151-156.

JAMES, P. L., CANNON, J., CRAWFORD, L., D'SOUZA, E., BARBER, C., COWMAN, S., COOKSON, W. O., MOFFATT, M. F. & CULLINAN, P. 2015. Molecular detection of mycobacterium avium in aerosolised metal working fluid is linked to a localised outbreak of extrinsic allergic alveolitis in factory workers. *American Journal of Respiratory and Critical Care Medicine,* 191.

KAGEN, S. L., FINK, J. N., SCHLUETER, D. P., KURUP, V. P. & FRUCHTMAN, R. B. 1981. Streptomyces albus: a new cause of hypersensitivity pneumonitis. *Journal of Allergy and Clinical Immunology,* 68**,** 295-9.

KAI, N., ISHII, H., IWATA, A., UMEKI, K., SHIRAI, R., MORINAGA, R., KISHI, K., TOKIMATSU, I., HIRAMATSU, K., YAMAGATA, E. & KADOTA, J. 2008. [Chronic hypersensitivity pneumonitis induced by Shiitake mushroom cultivation: case report and review of literature]. *Nihon Kokyuki Gakkai zasshi,* 46**,** 411-415.

KALAYCIOGLU, B. & BICAKOGLU, C. 2013. A case of hypersensitivity pneumonitis and imaging response to treatment. *Chest,* 144.

KAMM, Y. J., FOLGERING, H. T., VAN DEN BOGART, H. G. & COX, A. 1991. Provocation tests in extrinsic allergic alveolitis in mushroom workers. *Netherlands Journal of Medicine,* 38**,** 59-64.

KAWANE, H. & SOEJIMA, R. 1987. Hypersensitivity pneumonitis in a hairdresser. *Chest,* 92**,** 577-8.

KHOMENKO, A. G. & OZEROVA, L. V. 1996. [Occupation exogenous allergic alveolitis]. *Problemy Tuberkuleza***,** 72-75.

KHOMENKO, A. G., ZHALOLOV, Z., DMITRIEVA, L. I., OZEROVA, L. V. & IL'INA, I. N. 1989. [Clinical manifestations of extrinsic allergic alveolitis--a disease of tobacco growers]. *Klinicheskaia Meditsina,* 67**,** 61-65.

KIM, Y. J., HWANG, E. D., LEEM, A. Y., KANG, B. D., CHANG, S. Y., KIM, H. K., PARK, I. K., KIM, S. Y., KIM, E. Y., JUNG, J. Y., KANG, Y. A., PARK, M. S., KIM, Y. S., KIM, S. K., CHANG, J. & CHUNG, K. S. 2014. A case of occupational hypersensitivity pneumonitis associated with trichloroethylene. *Tuberculosis and Respiratory Diseases,* 76**,** 75-79.

KING, J., RICHARDSON, M., QUINN, A.-M., HOLME, J. & CHAUDHURI, N. 2017. Bagpipe lung a new type of interstitial lung disease? *Thorax,* 72**,** 380-382.

KIRKELEIT, J., SVANES, O., BERTELSEN, R. J., AASEN, T. B. & SVANES, C. 2017. Hypersensitivity pneumonitis in a fish-processing worker-use of specific inhalation challenge as a diagnostic tool. *European Respiratory Journal,* 50.

KISHIMOTO, N., MOURI, M., SAKURAI, S., NANBU, Y. & OHYA, N. 1993. [A case of hypersensitivity pneumonitis in Pholiota nameko's manufacturer]. *Nihon Kyobu Shikkan Gakkai zasshi,* 31**,** 275-9.

KOKKARINEN, J., TUKIAINEN, H. & TERHO, E. O. 1992. Severe farmer's lung following a workplace challenge. *Scandinavian Journal of Work, Environment and Health,* 18**,** 327-328.

KONISHI, K., MOURI, T., KOJIMA, Y., CHIDA, E., SUGAWARA, K., ABE, K., BANDO, T., ISHII, M. & TAMURA, M. 1994. Three cases of hypersensitivity pneumonitis caused by inhalation of spores of Pholiota nameko and the background of the disease. *Japanese Journal of Thoracic Diseases,* 32**,** 655-661.

KOSCHEL, D., HANDZHIEV, S., CARDOSO, C., ROLLE, A., HOLOTIUK, O. & HOFFKEN, G. 2011. Pneumomediastinum as a primary manifestation of chronic hypersensitivity pneumonitis. *Medical Science Monitor,* 17**,** CS152-CS155.

KOSCHEL, D., HOLFERT, J., ROLLE, A., HOLOTIUK, O. & HOFFKEN, G. 2012. Farmer's lung in a case after bullectomy. *International Archives of Allergy and Immunology,* 158**,** 313-316.

KUPELI, E., KARNAK, D., SAK, S. D. & KAYACAN, O. 2010. Hazards of the 'hard cash': Hypersensitivity pneumonitis. *Canadian Respiratory Journal,* 17**,** e102-e105.

LAL, A., AKHTAR, J., PINTO, S., GREWAL, H. & MARTIN, K. 2018. Recurrent Pulmonary Embolism and Hypersensitivity Pneumonitis Secondary to Aspergillus, in a Compost Plant Worker: Case Report and Review of Literature. *Lung,* 196**,** 553-560.

LANDER, F., JEPSEN, J. R. & GRAVESEN, S. 1988. Allergic alveolitis and late asthmatic reaction due to molds in the tobacco industry. *Allergy,* 43**,** 74-6.

LANSPA, M. J. & HATTON, N. D. 2014. Phanerochaete chrysosporium and granulomatous lung disease in a mulch gardener. *Respirology Case Reports,* 2**,** 7-9.

LHOUMEAU, A., PERNOT, J., GEORGES, M., DEVILLIERS, Y., CHARLES DALPHIN, J., CAMUS, P. & BONNIAUD, P. 2012. Hypersensitivity pneumonitis due to isocyanate exposure in an airbag "welder". *European respiratory review,* 21**,** 168-9.

LIIPPO, K. K., ANTTILA, S. L., TAIKINA-AHO, O., RUOKONEN, E.-L., TOIVONEN, S. T. & TUOMI, T. 1993. Hypersensitivity Pneumonitis and Exposure to Zirconium Silicate in a Young Ceramic Tile Worker. *American Review of Respiratory Disease,* 148**,** 1089-1092.

LINGENFELSER, A. & SENNEKAMP, J. 2010. [Extrinsic allergic alveolitis in a female podiatrist: Chiropody alveolitis]. *Allergologie,* 33**,** 573-574.

LOVERDOS, K., KARAHALIOU, I., ECONOMIDOU, E., GAGA, M. & KOLILEKAS, L. 2015. Hypersensitivity pneumonitis in a pleurotus mushroom grower. *Pneumon,* 28**,** 179-184.

LUNN, J. A. & HUGHES, D. T. 1967. Pulmonary hypersensitivity to the grain weevil. *British Journal of Industrial Medicine,* 24**,** 158-161.

MACKIEWICZ, B., SKORSKA, C., DUTKIEWICZ, J., MICHNAR, M., MILANOWSKI, J., PRAZMO, Z., KRYSINSKA-TRACZYK, E. & CISAK, E. 1999. Allergic alveolitis due to herb dust exposure. *Annals of Agricultural and Environmental Medicine,* 6**,** 167-170.

MACLEOD, I. M. & BROWN, W. R. 1982. Farmer's lung in an iron foundry. *Scottish Medical Journal,* 27**,** 154-7.

MAGNAVITA, N. 2013. Inadequate access to diagnostic resources: A case of unrecognised hypersensitivity pneumonitis. *Medicina del Lavoro,* 104**,** 67-72.

MALO, J. L. & ZEISS, C. R. 1982. Occupational hypersensitivity pneumonitis after exposure to diphenylmethane diisocyanate. *American Review of Respiratory Disease,* 125**,** 113-116.

MARCHISIO, V. F., SULOTTO, F., BOTTA, G. C., CHIESA, A., AIRAUDI, D. & ANASTASI, A. 1999. Aerobiological analysis in a salami factory: A possible case of extrinsic allergic alveolitis by Penicillium camembertii. *Medical Mycology,* 37**,** 285-289.

MARIA, Y., FILLIARD, E. & HENNINGER, J. F. 1992. [Allergic alveolitis due to colophony]. *Revue des Maladies Respiratoires,* 9**,** 472-3.

MARLAND, P., TABART, J., BERSAY, C., PENINOU-GALL, G., BORY, J., LABARRE, C. & ELBAZE, P. 1982. [Mushroom growers lung: Apropos of 3 cases]. *Poumon et le Coeur,* 38**,** 371-376.

MARTIN-GARCIA, C., HINOJOSA, M., PORCEL, S., LEON, F. & BERGES, P. 2003. Corn-induced hypersensitivity pneumonitis. *Allergy,* 58**,** 534-535.

MARX JR, J. J., GUERNSEY, J., EMANUEL, D. A., MERCHANT, J. A., MORGAN, D. P. & KRYDA, M. 1990. Cohort studies of immunologic lung disease among Wisconsin dairy farmers. *American Journal of Industrial Medicine,* 18**,** 263-268.

MATSUSHIMA, H., TAKAYANAGI, N., TOKUNAGA, D., MAENO, Y., SATO, N., KURASHIMA, K., UBUKATA, M., YANAGISAWA, T., SUGITA, Y., KAWABATA, Y. & KANAZAWA, M. 2003. [A case of combined hypersensitivity pneumonitis and bronchial asthma due to isocyanate (MDI)]. *Nihon Kokyuki Gakkai zasshi,* 41**,** 760-765.

MAYER, K. H., LEITNER, L., MOSENBACHER, A. & ECKMAYR, J. 1990. [Alveolitis caused by furnishing a medical office]. *Pneumologie,* 44**,** 901-2.

MDER, I., LIEBETRAU, G. & TREUTLER, D. 1988. [Exogenous allergic alveolitis in a fruit refrigeration worker]. *Zeitschrift fur die Gesamte Innere Medizin und Ihre Grenzgebiete,* 43**,** 221-2.

MEHTA, P., WILLS, P., KOHLI, S. & DUBREY, S. 2008. Pigeon fanciers lung: a case report. *Cases Journal,* 1**,** 37.

MERGET, R., MARCZYNSKI, B., CHEN, Z., REMBERGER, K., RAULF-HEIMSOTH, M., WILLROTH, P. O. & BAUR, X. 2002. Haemorrhagic hypersensitivity pneumonitis due to naphthylene-1,5-diisocyanate. *European Respiratory Journal,* 19**,** 377-380.

MERGET, R., SANDER, I., ROZYNEK, P., RAULF-HEIMSOTH, M. & BRUENING, T. 2008. Occupational hypersensitivity pneumonitis due to molds in an onion and potato sorter. *American Journal of Industrial Medicine,* 51**,** 117-119.

METERSKY, M. L., BEAN, S. B., MEYER, J. D., MUTAMBUDZI, M., BROWN-ELLIOTT, B. A., WECHSLER, M. E. & WALLACE, R. J., JR. 2010. Trombone player's lung: a probable new cause of hypersensitivity pneumonitis. *Chest,* 138**,** 754-6.

METZGER, F., HACCURIA, A., REBOUX, G., NOLARD, N., DALPHIN, J.-C. & DE VUYST, P. 2010. Hypersensitivity pneumonitis due to molds in a saxophone player. *Chest,* 138**,** 724-6.

MIEDINGER, D., MALO, J. L., CARTIER, A. & LABRECQUE, M. 2009. Malt can cause both occupational asthma and allergic alveolitis. *Allergy,* 64**,** 1228-1229.

MILANOWSKI, J., DUTKIEWICZ, J., POTOCZNA, H., KUS, L. & URBANOWICZ, B. 1998. Allergic alveolitis among agricultural workers in eastern Poland: a study of twenty cases. *Annals of Agricultural and Environmental Medicine : AAEM,* 5**,** 31-43.

MITANI, M., SATOH, K., KOBAYASHI, T., KAWASE, Y., HOSOKAWA, N., TAKASHIMA, H., KOBAYASHI, S. & TANABE, M. 1995. Hypersensitivity pneumonitis in a pearl nucleus worker. *Journal of Thoracic Imaging,* 10**,** 134-137.

MIYAZAKI, H., GEMMA, H., KOSHIMIZU, N., SATO, M., ITO, I., SUDA, T., CHIDA, K. & NAKAMURA, H. 2003. [Hypersensitivity pneumonitis induced by Pleurotus eryngii spores--a case report]. *Nihon Kokyuki Gakkai zasshi,* 41**,** 827-33.

MIYAZAKI, H., GEMMA, H., UEMURA, K., ONO, T., MASUDA, M., SANO, T., SATO, M., KOSHIMIZU, N., SUDA, T. & CHIDA, K. 2004. [Hypersensitivity pneumonitis induced by Aspergillus niger--a case report]. *Nihon Kokyuki Gakkai zasshi,* 42**,** 676-681.

MIYAZAKI, H., HIRATA, T., SHIMANE, S., MORITA, S., CHIHARA, K., ENOMOTO, N., SUDA, T. & CHIDA, K. 2006. [A case of hypersensitivity pneumonitis caused by zinc fume]. *Nihon Kokyuki Gakkai zasshi,* 44**,** 985-9.

MOLDOVAN, H. R., IONOVICI, N., NECHITA, F., HORVATH, E., IANOSI, E. S., PAPP, E. G., POPOVICIU, H. V., JIMBOREAN, G., MOLDOVAN, G., VLASIU, M. A. & SZASZ, S. 2019. A rare association of cutaneous leukocytoclastic angiitis (hypersensitivity vasculitis) and hypersensitivity pneumonia (extrinsic allergic alveolitis) in a pigeon breeder - case report and literature review. *Romanian Journal of Morphology and Embryology,* 60**,** 325-331.

MONIODIS, A., HAMILTON, T., RACILA, E., COCKRILL, B. & MCCUNNEY, R. 2015. Hypersensitivity pneumonitis in a high school teacher. *Occupational Medicine,* 65**,** 598-600.

MORAGA-MCHALEY, S. A., LANDEN, M., KRAPFL, H. & SEWELL, C. M. 2013. Hypersensitivity pneumonitis with Mycobacterium avium complex among spa workers. *International Journal of Occupational and Environmental Health,* 19**,** 55-61.

MORAL, A. J., ARIAS, J., GARCIA, M. A., ABENGOZAR, R., PEREZ-CARRAL, C. & SENENT, C. J. 1994. [Extrinsic allergic alveolitis caused by Penicillium frequentans. Review and presentation of a case]. *Archivos de Bronconeumologia,* 30**,** 462-4.

MORELL, F., CRUZ, M. J., GOMEZ, F. P., RODRIGUEZ-JEREZ, F., XAUBET, A. & MUNOZ, X. 2011. Chacineros lung - hypersensitivity pneumonitis due to dry sausage dust. *Scandinavian Journal of Work, Environment and Health,* 37**,** 349-356.

MORELL, F., GOMEZ, F., URESANDI, F., GONZALEZ, A., SUAREZ, I. & RODRIGO, M. J. 1995. Espartosis. A new type of extrinsic allergic alveolitis among construction plasterers. *Medicina clinica,* 105**,** 19-23.

MORELL, F., ROGER, A., CRUZ, M. J., MUNOZ, X. & RODRIGO, M. J. 2003. Suberosis: Clinical study and new etiologic agents in a series of eight patients. *Chest,* 124**,** 1145-1152.

MORENO-ANCILLO, A., DOMINGUEZ-NOCHE, C., GIL-ADRADOS, A. C. & COSMES, P. M. 2003. Familiar presentation of occupational hypersensitivity pneumonitis caused by aspergillus-contaminated esparto dust. *Allergologia et Immunopathologia,* 31**,** 294-296.

MORENO-ANCILLO, A., DOMINGUEZ-NOCHE, C., GIL-ADRADOS, A. C. & COSMES, P. M. 2004. Hypersensitivity pneumonitis due to occupational inhalation of fungi-contaminated corn dust. *Journal of Investigational Allergology and Clinical Immunology,* 14**,** 165-167.

MORENO-ANCILLO, A., PADIAL, M. A., LOPEZ-SERRANO, M. C. & GRANADO, S. 1997. Hypersensitivity pneumonitis due to inhalation of fungi-contaminated esparto dust in a plaster worker. *Allergy and Asthma Proceedings,* 18**,** 355-357.

MORI, S., NAKAGAWA-YOSHIDA, K., TSUCHIHASHI, H., KOREEDA, Y., KAWABATA, M., NISHIURA, Y., ANDO, M. & OSAME, M. 1998. Mushroom worker's lung resulting from indoor cultivation of Pleurotus osteatus. *Occupational Medicine,* 48**,** 465-468.

MOURA, S. & PINELO, E. 2011. Interstitial lung disease: About two cases. *European Journal of Internal Medicine,* 22**,** S63-S64.

MULLER-WENING, D., RENCK, T. & NEUHAUSS, M. 1999. Fuel chip-induced hypersensitivity pneumonitis, severe reaction after work-related challenge. *Pneumologie,* 53**,** 364-368.

MURAKAMI, M., KAWABE, K., HOSOI, Y., HOJO, S., DOBASHI, K., IRIUCHIJIMA, T., NAKAZAWA, T. & MORI, M. 1997. Decreased pulmonary perfusion in hypersensitivity pneumonitis caused by Shiitake mushroom spores. *Journal of Internal Medicine,* 241**,** 85-88.

NAHATA, A. & ESSEN, S. V. 2013. Occupational hypersensitivity pneumonitis after pyrethroid insecticide exposure. *Chest,* 144.

NAKAMURA, Y., FUJIMOTO, H., UETANI, K., SUMDA, T., HIGASHIMOTO, Y., FUNASAKO, M. & OHATA, M. 1995. A case of chronic hypersensitivity pneumonitis due to long term exposure to toluene diisocyanate. *Japanese Journal of Thoracic Diseases,* 33**,** 429-432.

NAKAZAWA, T. & TOCHIGI, T. 1989. Hypersensitivity pneumonitis due to mushroom (Pholiota nameko) spores. *Chest,* 95**,** 1149-51.

NAKAZAWA, T. & UMEGAE, Y. 1990. Sericulturist's lung disease: hypersensitivity pneumonitis related to silk production. *Thorax,* 45**,** 233-4.

NEDDAM, P., POMMIER DE SANTI, P., CARBONNEL, M. C., DOR, P., GARBE, A. M., STEINMETZ, M. D., RASCOL, J. P. & RIBEROT, H. 1998. Extrinsic allergic alveolitis in a wine trading firm. Suberosis? *Revista Portuguesa de Imunoalergologia.,* 59**,** 110-113.

NEFEDOV, V. B., POPOVA, L. A. & ZHALOLOV, Z. Z. 1991. [Lung function in tobacco growers suffering from exogenous allergic alveolitis]. *Terapevticheskii Arkhiv,* 63**,** 124-126.

NORDNESS, M. E., ZACHARISEN, M. C., SCHLUETER, D. P. & FINK, J. N. 2003. Occupational lung disease related to Cytophaga endotoxin exposure in a nylon plant. *Journal of Occupational and Environmental Medicine,* 45**,** 385-392.

NOZAWA, S., SATO, T., SASAGAWA, M., SUZUKI, E., KIOI, S. & ARAKAWA, M. 1989. [A case of hypersensitivity pneumonitis due to isocyanate (TDI)]. *Nihon Kyobu Shikkan Gakkai zasshi,* 27**,** 1335-41.

O'BRIEN, D. M. 2003. Aerosol Mapping of a Facility with Multiple Cases of Hypersensitivity Pneumonitis: Demonstration of Mist Reduction and a Possible Dose/Response Relationship. *Applied Occupational and Environmental Hygiene,* 18**,** 947-952.

O'CONNELL, M. A., PLUSS, J. L., SCHKADE, P., HENRY, A. R. & GOODMAN, D. L. 1995. Rhizopus-induced hypersensitivity pneumonitis in a tractor driver. *Journal of Allergy and Clinical Immunology,* 95**,** 779-80.

OH, M. N., CHO, M. J., BAEK, H. K., CHO, K. S., KANG, J. H., KIM, Y. & KWAK, J. Y. 2008. A case of hypersensitivity pneumonitis in an automobile paint sprayer. *Tuberculosis and Respiratory Diseases,* 65**,** 541-545.

OMURA, H., KAJIKI, A., IKEGAME, S., AONO, A., MITARAI, S. & KITAHARA, Y. 2012. [Mycobacterium immunogenum isolated from a metal worker in Japan]. *Kekkaku : [Tuberculosis],* 87**,** 341-344.

ORRIOLS, R., ALIAGA, J. L. I., ANTO, J. M., FERRER, A., HERNANDEZ, A., RODRIGO, M. J. & MORELL, F. 1997. High prevalence of mollusc shell hypersensitivity pneumonitis in nacre factory workers. *European Respiratory Journal,* 10**,** 780-786.

ORRIOLS, R., MANRESA, J. M., ALIAGA, J. L., CODINA, R., RODRIGO, M. J. & MORELL, F. 1990. Mollusk shell hypersensitivity pneumonitis. *Annals of Internal Medicine,* 113**,** 80-81.

PAL, T. M., DE MONCHY, J. G. R., GROOTHOFF, J. W. & POST, D. 1997. The clinical spectrum of humidifier disease in synthetic fiber plants. *American Journal of Industrial Medicine,* 31**,** 682-692.

PARIS, C., HERIN, F., REBOUX, G., PENVEN, E., BARRERA, C., GUIDAT, C. & THAON, I. 2015. Working with argan cake: A new etiology for hypersensitivity pneumonitis. *BMC Pulmonary Medicine,* 15**,** 18.

PARTRIDGE, S. J., PEPPERELL, J. C. T., FORRESTER-WOOD, C., IBRAHIM, N. B. N., RAYNAL, A. & SWINBURN, C. R. 2004. Pheasant rearer's lung. *Occupational Medicine,* 54**,** 500-503.

PATTERSON, R., SOMMERS, H. & FINK, J. N. 1974. Farmer's lung following inhalation of Aspergillus flavus growing in mouldy corn. *Clinical Allergy,* 4**,** 79-86.

PIIRILA, P., KESKINEN, H., ANTTILA, S., HYVONEN, M., PFAFFLI, P., TUOMI, T., TUPASELA, O., TUPPURAINEN, M. & NORDMAN, H. 1997. Allergic alveolitis following exposure to epoxy polyester powder paint containing low amounts (<1%) of acid anhydrides. *European Respiratory Journal,* 10**,** 948-951.

PIMENTEL, J. C. 1970. Furrier's lung. *Thorax,* 25**,** 387.

PIMENTEL, J. C., AVILA, R. & LOURENCO, A. G. 1975. Respiratory disease caused by synthetic fibres: a new occupational disease. *Thorax,* 30**,** 204-19.

PIMENTEL, J. C. & MARQUES, F. 1969. "Vineyard sprayer's lung": a new occupational disease. *Thorax,* 24**,** 678-688.

POLIAKOVA, I. N. 1992. [A case of extrinsic allergic alveolitis in a shoe factory worker]. *Gigiena Truda i Professional'nye Zabolevaniia***,** 38-40.

PREISSER, A. M. & HARTH, V. 2017. Organic waste collectors - risk of exogenous allergic alveolitis. *Allergologie,* 40**,** 303-305.

PU, C. Y., RASHEED, M. R. H. A., SEKOSAN, M. & SHARMA, V. 2017. Pet Groomer's Lung: A novel occupation related hypersensitivity pneumonitis related to pyrethrin exposure in a pet groomer. *American Journal of Industrial Medicine,* 60**,** 141-145.

QUIRCE, S., VANDENPLAS, O., CAMPO, P., CRUZ, M. J., DE BLAY, F., KOSCHEL, D., MOSCATO, G., PALA, G., RAULF, M., SASTRE, J., SIRACUSA, A., TARLO, S. M., WALUSIAK-SKORUPA, J. & CORMIER, Y. 2016. Occupational hypersensitivity pneumonitis: An EAACI position paper. *Allergy,* 71**,** 765-779.

RASK-ANDERSEN, A. 1989. Allergic alveolitis in Swedish farmers. *Upsala Journal of Medical Sciences,* 94**,** 271-285.

RASK-ANDERSEN, A., LAND, C. J., ENLUND, K. & LUNDIN, A. 1994. Inhalation fever and respiratory symptoms in the trimming Department of Swedish sawmills. *American Journal of Industrial Medicine,* 25**,** 65-67.

REBOUX, G., PIARROUX, R., MAUNY, F., MADROSZYK, A., MILLON, L., BARDONNET, K. & DALPHIN, J.-C. 2001. Role of Molds in Farmer's Lung Disease in Eastern France. *American Journal of Respiratory and Critical Care Medicine,* 163**,** 1534-1539.

REED, C. E., SWANSON, M. C., LOPEZ, M., FORD, A. M., MAJOR, J., WITMER, W. B. & VALDES, T. B. 1983. Measurement of IgG antibody and airborne antigen to control an industrial outbreak of hypersensitivity pneumonitis. *Journal of Occupational Medicine,* 25**,** 207-10.

REHEDA, M. S. 1994. [The characteristics of the protein and lipid metabolic indices of poultry farmers with extrinsic allergic alveolitis]. *Fiziolohichnyi Zhurnal,* 40**,** 118-20.

REYNAUD, C., VODOZ, J. F., BERNSTEIN, M., NERBOLLIER, G., RICHARDET, C. & POLLA, B. S. 1992. [Sausage and extrinsic allergic alveolitis: A new occupational disease in Switzerland]. *Sozial- und Praventivmedizin,* 37**,** 263-268.

RIARIO SFORZA, G. G. & MARINOU, A. 2017. Hypersensitivity pneumonitis: A complex lung disease. *Clinical and Molecular Allergy,* 15**,** 6.

RICCO, M., VIGNALI, A. & PESCI, M. 2019. Hypersensitivity pneumonia and HIV infection in occupational settings: a case report from northern Italy. *Acta bio-medica : Atenei Parmensis,* 90**,** 331-335.

RICHERSON, H. B., BERNSTEIN, I. L., FINK, J. N., HUNNINGHAKE, G. W., NOVEY, H. S., REED, C. E., SALVAGGIO, J. E., SCHUYLER, M. R., SCHWARTZ, H. J. & STECHSCHULTE, D. J. 1989. Guidelines for the clinical evaluation of hypersensitivity pneumonitis. Report of the Subcommittee on Hypersensitivity Pneumonitis. *Journal of Allergy and Clinical Immunology,* 84**,** 839-843.

RIDDLE, H. F., CHANNELL, S., BLYTH, W., WEIR, D. M., LLOYD, M., AMOS, W. M. & GRANT, I. W. 1968. Allergic alveolitis in a maltworker. *Thorax,* 23**,** 271-80.

RIVERO, M. G., BASILE, L. M., SALVATORE, A. J., FRIDLENDER, H. & MAXIT, M. 1999. [Salami worker's lung]. *Medicina,* 59**,** 367-9.

ROBERTSON, A. S., BURGE, P. S., WIELAND, G. A. & CARMALT, M. H. 1987. Extrinsic allergic alveolitis caused by a cold water humidifier. *Thorax,* 42**,** 32-7.

ROBERTSON, W., ROBERTSON, A. S., BURGE, C. B. S. G., MOORE, V. C., JAAKKOLA, M. S., DAWKINS, P. A., BURD, M., RAWBONE, R., GARDNER, I., KINOULTY, M., CROOK, B., EVANS, G. S., HARRIS-ROBERTS, J., RICE, S. & BURGE, P. S. 2007. Clinical investigation of an outbreak of alveolitis and asthma in a car engine manufacturing plant. *Thorax,* 62**,** 981-990.

ROUSSEL, S., REBOUX, G., ROGNON, B., MONOD, M., GRENOUILLET, F., QUADRONI, M., FELLRATH, J. M., AUBERT, J. D., DALPHIN, J. C. & MILLON, L. 2010. Comparison of three antigenic extracts of Eurotium amstelodami in serological diagnosis of farmer's lung disease. *Clinical and Vaccine Immunology,* 17**,** 160-167.

ROUSSEL, S., ROGNON, B., BARRERA, C., REBOUX, G., SALAMIN, K., GRENOUILLET, F., THAON, I., DALPHIN, J.-C., TILLIE-LEBLOND, I., QUADRONI, M., MONOD, M. & MILLON, L. 2011. Immuno-reactive proteins from Mycobacterium immunogenum useful for serodiagnosis of metalworking fluid hypersensitivity pneumonitis. *International Journal of Medical Microbiology,* 301**,** 150-6.

RUDRAPPA, M. & KOKATNUR, L. 2017. Hot Tub Lung: An Intriguing Diffuse Parenchymal Lung Disease. *Ghana medical journal,* 51**,** 143-147.

SAAK, A. & STRESEMANN, E. 1994. Exogenous allergic alveolitis and unusual manifestation of eczema due to isocyanate. *Arbeitsmedizin Sozialmedizin Umweltmedizin,* 29**,** 256-259.

SAIKAI, T., TANAKA, H., FUJI, M., SUGAWARA, H., TAKEYA, I., TSUNEMATSU, K. & ABE, S. 2002. Hypersensitivity pneumonitis induced by the spore of Pleurotus Eryngii (Eringi). *Internal Medicine,* 41**,** 571-573.

SAKURAI, M., KINOSITA, K., KOBAYASHI, Y., NISHI, Y., NOZAWA, M., KAWASAKI, M., TABE, K., NAGATA, M., KURAMITU, K., SAKAMOTO, Y. & SHIMIZU, Y. 2001. [Hypersensitivity pneumonitis caused by a factory humidifier. A case report]. *Nihon Kokyuki Gakkai zasshi,* 39**,** 190-4.

SANDERSON, W., KULLMAN, G., SASTRE, J., OLENCHOCK, S., O'CAMPO, A., MUSGRAVE, K. & GREEN, F. 1992. Outbreak of hypersensitivity pneumonitis among mushroom farm workers. *American Journal of Industrial Medicine,* 22**,** 859-872.

SANO, T., GEMMA, H., SATOU, M., ONO, T., ATSUMI, E., ITO, I., CHIDA, K., SUDA, T., NAKAMURA, H., TOYOSHIMA, M. & SHIRAI, T. 2004. A Case of Hypersensitivity Pneumonitis in a Tea Bag Manufacturer Employee. *Japanese Journal of Chest Diseases,* 63**,** 393-398.

SARTORELLI, P., D’HAUW G., SPINA D., VOLTERRANI L., MAZZEI M.A. 2020. A case of hypersensitivity pneumonitis in a worker exposed to terephthalic acid in the production of polyethylene terephthalate. *International Journal of Occupational Medicine and Environmental Health*, 33, 119-123.

SATOH, M., JINUSHI, E., WAKABAYASHI, O., YOSHIDA, F. & ARAYA, Y. 2011. [Chronic and acute hypersensitivity pneumonitis in a dairy farmer and his son]. *Arerugi,* 60**,** 43-50.

SCHERPEREEL, A., TILLIE-LEBLOND, I., POMMIER DE SANTI, P. & TONNEL, A. B. 2004. Exposure to methyl methacrylate and hypersensitivity pneumonitis in dental technicians. *Allergy: European Journal of Allergy and Clinical Immunology,* 59**,** 890-892.

SCHLEGEL, V., LIEBETRAU, G. & POHL, W. D. 1990. [Swine breeder's lung--a form of exogenous allergic alveolitis]. *Zeitschrift fur Erkrankungen der Atmungsorgane,* 174**,** 143-148.

SCHLUETER, D. P. 1973. "Cheesewasher's Disease": A New Occupational Hazard? *Annals of Internal Medicine,* 78**,** 606-606.

SCHLUETER, D. P., FINK, J. N. & HENSLEY, G. T. 1972. Wood-pulp workers' disease: a hypersensitivity pneumonitis caused by Alternaria. *Annals of Internal Medicine,* 77**,** 907-14.

SCHNEIDER, J., FREITAG, F. & RODELSPERGER, K. 1994. Exogenous allergic alveolitis (N. 4201 BeKV) caused by exposure to zirconium at the workplace. *Arbeitsmedizin Sozialmedizin Umweltmedizin,* 29**,** 382-385.

SCHREIBER, J., KNOLLE, J., SENNEKAMP, J., SCHULZ, K. T., HAHN, J. U., HERING, K. G., RAULF-HEIMSOTH, M. & MERGET, R. 2008. Sub-acute occupational hypersensitivity pneumonitis due to low-level exposure to diisocyanates in a secretary. *European Respiratory Journal,* 32**,** 807-811.

SCHREIBER, J., MULLER, E., BECKER, W. M., ZABEL, P., SCHLAAK, M. & AMTHOR, M. 1998. [Spinach powder-induced exogenous allergic alveolitis]. *Pneumologie,* 52**,** 61-5.

SCHULTE, W., JOEST, M. & SENNEKAMP, J. 2008. Exogen-allergic alveolitis caused by a biological detergent. *Allergologie,* 31**,** 487-492.

SHANMUGAPRIYA, K., DHANASEKAR, T., HARIPRASAD, B. & VIJAYALAKSHMI, T. 2013. Unusual causes of hypersensitivity pneumonitis: Two cases. *Lung India,* 30**,** S57-S58.

SHARMA, B., SINGHA, A., CHAUDHARY, O., SAH, B., NAT, A. & MANTA, D. 2013. Hypersensitivity pneumonitis in a occupational nail worker. *Critical Care Medicine,* 41**,** A336.

SHELTON, B. G., FLANDERS, W. D. & MORRIS, G. K. 1999. Mycobacterium sp. as a possible cause of hypersensitivity pneumonitis in machine workers. *Emerging Infectious Diseases,* 5**,** 270-3.

SHEPHERD, G. M., MICHELIS, M. A., MACRIS, N. T. & SMITH, J. P. 1989. Hypersensitivity pneumonitis in an orchid grower associated with sensitivity to the fungus Cryptostroma corticale. *Annals of Allergy,* 62**,** 522-525.

SHIVANTHAN, M. C. & WIJESIRIWARDENA, B. 2011. Bronchoalveolar carcinoma on a background of chronic extrinsic allergic alveolitis in a spice miller - A case report. *Respiratory Medicine CME,* 4**,** 119-120.

SOLLEY, G. O. & HYATT, R. E. 1980. Hypersensitivity pneumonitis induced by Penicillium species. *Journal of Allergy and Clinical Immunology,* 65**,** 65-70.

SOUMAGNE, T., REBOUX, G., DEGANO, B. & DALPHIN, J. C. 2016. Hypersensitivity pneumonitis in a beautician. *American journal of industrial medicine,* 59**,** 1041-1045.

STORMS, W. W. 1978. Occupational hypersensitivity lung disease. *Journal of Occupational Medicine,* 20**,** 823-824.

STRAND, R. D., NEUHAUSER, E. B. D. & SORNBERGER, C. F. 1967. Lycoperdonosis. *New England Journal of Medicine,* 277**,** 89-91.

SUGA, M. & SUKOH, N. 2002. A case of subacute budgerigar breeder's lung presenting a marked elevation of serum SP-D and KL-6. *Japanese Journal of Chest Diseases,* 61**,** 1022-1026.

SUZUKI, K., TANAKA, H., SUGAWARA, H., SAITO, Y., KOBA, H., TSUNEMATSU, K. & ABE, S. 2001. Chronic hypersensitivity pneumonitis induced by Shiitake mushroom spores associated with lung cancer. *Internal medicine,* 40**,** 1132-5.

SVANES, O., KIRKELEIT, J., AASEN, T. B., SVENDSEN, L., STORAAS, T., BERTELSEN, R. & SVANES, C. 2014. Hypersensitivity pneumonitis in hairdressers. *European Respiratory Journal,* 44.

TAHA BEKCI, T., CALIK, M., CALIK, S. G. & ESME, H. 2014. Oyster mushrooms (pleurotus ostreatus) caused hypersensitivity pneumonitis: Mushroom worker's lung. *European Respiratory Journal,* 44.

TAJIMA, S., KON, H., OSHIKAWA, K., BANDO, M., OHNO, S. & SUGIYAMA, Y. 2003. Hypersensitivity pneumonitis induced by Konjak flour and powdered Hijikia fusiforme. *Internal Medicine,* 42**,** 846-849.

TAKAKU, Y., TAKAYANAGI, N., MINAGAWA, S., TSUCHIYA, Y., HIJIKATA, N., HARA, K., YAMAJI, T., TOKUNAGA, D., SAITO, H., UBUKATA, M., KURASHIMA, K., YANAGISAWA, T., SUGITA, Y. & KAWABATA, Y. 2009. [Hypersensitivity pneumonitis induced by Hypsizigus marumoreus]. *Nihon Kokyuki Gakkai zasshi,* 47**,** 881-889.

TANAKA, H., SAIKAI, T., SUGAWARA, H., TSUNEMATSU, K., TAKEYA, I., KOBA, H., MATSUURA, A., IMAI, K. & ABE, S. 2001. Three-year follow-up study of allergy in workers in a mushroom factory. *Respiratory Medicine,* 95**,** 943-948.

TANAKA, H., SUGAWARA, H., SAIKAI, T., TSUNEMATSU, K., TAKAHASHI, H. & ABE, S. 2000. Mushroom worker's lung caused by spores of Hypsizigus marmoreus (Bunashimeji): Elevated serum surfactant protein D levels. *Chest,* 118**,** 1506-1509.

TANAKA, H., TSUNEMATSU, K., NAKAMURA, N., SUZUKI, K., TANAKA, N., TAKEYA, I., SAIKAI, T. & ABE, S. 2004. Successful treatment of hypersensitivity pneumonitis caused by Grifola frondosa (Maitake) mushroom using a HFA-BDP extra-fine aerosol. *Internal Medicine,* 43**,** 737-740.

TANAKA, Y., SHIRAI, T., ENOMOTO, N., ASADA, K., OYAMA, Y. & SUDA, T. 2016. Occupational hypersensitivity pneumonitis in a green tea manufacturer. *Respirology Case Reports,* 4**,** e00152.

TANIGUCHI, H., MIWA, T., ABO, H., MIYAZAWA, H., NOTO, H., UCHIYAMA, A., MIWA, A. & IZUMI, S. 2004. A case of hypersensitivity pneumonitis caused by Lyophyllum karst. *Japanese Journal of Allergology,* 53**,** 696-699.

TANIOS, M. A., EL GAMAL, H., ROSENBERG, B. J. & HASSOUN, P. M. 2004. Can we still miss tetrachloroethylene-induced lung disease? The emperor returns in new clothes. *Respiration,* 71**,** 642-645.

TERHO, E. O., HUSMAN, K., KOTIMAA, M. & SJOBLOM, T. 1980. Extrinsic allergic alveolitis in a sawmill worker. A case report. *Scandinavian Journal of Work, Environment & Health,* 6**,** 153-7.

THOMAS, C., BIZIEUX-THAMINY, A., GAGNADOUX, F., GOURDIER, A. L., URBAN, T. & RACINEUX, J. L. 2005. [An unusual cause of pulmonary cysts]. *Revue des Maladies Respiratoires,* 22**,** 313-316.

THOMAS, H. 1995. [Exogenous allergic alveolitis: 22 year follow-up of malt worker's lung]. *Pneumologie,* 49**,** 32-4.

THORN, A., LEWNE, M. & BELIN, L. 1996. Allergic alveolitis in a school environment. *Scandinavian Journal of Work, Environment & Health,* 22**,** 311-4.

TILLIE-LEBLOND, I., GRENOUILLET, F., REBOUX, G., ROUSSEL, S., CHOURAKI, B., LORTHOIS, C., DALPHIN, J. C., WALLAERT, B. & MILLON, L. 2011. Hypersensitivity pneumonitis and metalworking fluids contaminated by mycobacteria. *European Respiratory Journal,* 37**,** 640-647.

TJALVIN, G., MIKKELSEN K.E., APELSETH, T.O., HOLLUND, B.E., SVANES, C., VAN DO,T. 2020. Hypersensitivity Pneumonitis in Farmers: Improving Etiologic Diagnosis to Optimize Counselling. *Journal of Agromedicine.* 25, 65-72.

TJALVIN, G., SVANES, O., BERTELSEN, R. J., HOLLUND, B. E., AASEN, T. B., SVANES, C. & KIRKELEIT, J. 2018. Hypersensitivity pneumonitis in fish processing workers diagnosed by inhalation challenge. *European Respiratory Journal Open Research,* 4.

TORRES JIMENEZ, C., PERELLO PERELLO, S. & RODRIGUEZ QUINTERO, J. 1979. Extrinsic allergic alveolitis caused by bagassosis. *Revista Cubana de Medicina,* 18**,** 263-276.

TRIPATHI, A. & GRAMMER, L. C. 2001. Extrinsic allergic alveolitis from a proteolytic enzyme. *Annals of Allergy, Asthma and Immunology,* 86**,** 425-427.

TSUCHIYA, Y., SHIMOKATA, K., OHARA, H., NISHIWAKI, K. & KINO, T. 1993. Hypersensitivity pneumonitis in a soy sauce brewer caused by Aspergillus oryzae. *Journal of Allergy and Clinical Immunology,* 91**,** 688-689.

TSUSHIMA, K., FUJIMOTO, K., YOSHIKAWA, S., KAWAKAMI, S., KOIZUMI, T. & KUBO, K. 2005. Hypersensitivity pneumonitis due to Bunashimeji mushrooms in the mushroom industry. *International Archives of Allergy and Immunology,* 137**,** 241-248.

TSUSHIMA, K., FURUYA, S., YOSHIKAWA, S., YASUO, M., YAMAZAKI, Y., KOIZUMI, T., FUJIMOTO, K. & KUBO, K. 2006. Therapeutic effects for hypersensitivity pneumonitis induced by Japanese mushroom (Bunashimeji). *American Journal of Industrial Medicine,* 49**,** 826-835.

TSUSHIMA, K., HONDA, T. & KUBO, K. 2000a. [A case of hypersensitivity pneumonitis caused by Strophariaceae]. *Nihon Kokyuki Gakkai zasshi,* 38**,** 536-539.

TSUSHIMA, K., HONDA, T. & KUBO, K. 2000b. [Hypersensitivity pneumonitis caused by Lyophyllum aggregatum in two sisters]. *Nihon Kokyuki Gakkai zasshi,* 38**,** 599-604.

QUIRCE, S., FERNÀNDEZ-NIETO, M., DE GÓRGOLAS, M., RENEDO, G., CARNÉS, J. & SASTRE, J. 2004. Hypersensitivity pneumonitis caused by triglycidyl isocyanurate. *Allergy,* 59**,** 1128-1128.

UNSAL, E., CANBAKAN, S., OFLUOGLU, R., ERTURK, A., CAPAN, N. & GULER, M. 2014. Hypersensitivity pneumonitis due to glue inhalation. *Respiratory Case Reports,* 3**,** 86-89.

USUI, Y., AIDA, H., KIMULA, Y., MIURA, H., TAKAYAMA, S. & NAKAYAMA, M. 1992. Hypersensitivity pneumonitis induced by hexamethylene diisocyanate. *Internal Medicine,* 31**,** 912-6.

VALBUENA, G., SAPORITI, A. M. & BUSTOS, C. 1993. [Thermoactinomyces vulgaris as a cause of extrinsic allergic alveolitis]. *Allergologia et immunopathologia,* 21**,** 229-232.

VAN ASSENDELFT, A., FORSEN, K. O., KESKINEN, H. & ALANKO, K. 1979. Humidifier-associated extrinsic allergic alveolitis. *Scandinavian Journal of Work, Environment and Health,* 5**,** 35-41.

VAN HEEMST, R. C., SANDER, I., ROOYACKERS, J., DE JONG, L., DJAMIN, R. S., AERTS, J. G. & BELDERBOS, H. N. A. 2009. Hypersensitivity pneumonitis caused by occupational exposure to phytase. *European Respiratory Journal,* 33**,** 1507-1509.

VAN TOORN, D. W. 1970. Coffee worker's lung. A new example of extrinsic allergic alveolitis. *Thorax,* 25**,** 399-405.

VEILLETTE, M., CORMIER, Y., ISRAEL-ASSAYAQ, E., MERIAUX, A. & DUCHAINE, C. 2006. Hypersensitivity pneumonitis in a hardwood processing plant related to heavy mold exposure. *Journal of Occupational and Environmental Hygiene,* 3**,** 301-7.

VILLAR, A., MUNOZ, X., CRUZ, M. J. & MORELL, F. 2009. [Hypersensitivity pneumonitis caused by Mucor species in a cork worker]. *Archivos de Bronconeumologia,* 45**,** 405-7.

VINCKEN, W. & ROELS, P. 1984. Hypersensitivity pneumonitis due to Aspergillus fumigatus in compost. *Thorax,* 39**,** 74.

VOLKMAN, K. K., MERRICK, J. G. & ZACHARISEN, M. C. 2006. Yacht-maker's lung: A case of hypersensitivity pneumonitis in yacht manufacturing. *Wisconsin Medical Journal,* 105**,** 47-50.

WALKER, C. L., GRAMMER, L. C., SHAUGHNESSY, M. A., DUFFY, M., STOLTZFUS, V. D. & PATTERSON, R. 1989. Diphenylmethane diisocyanate hypersensitivity pneumonitis: A serologic evaluation. *Journal of Occupational Medicine,* 31**,** 315-319.

WALTERS, G. I., MOKHLIS, J. M., MOORE, V. C., ROBERTSON, A. S., BURGE, G. A., BHOMRA, P. S. & BURGE, P. S. 2019. Characteristics of hypersensitivity pneumonitis diagnosed by interstitial and occupational lung disease multi-disciplinary team consensus. *Respiratory Medicine,* 155**,** 19-25.

WALTERS, G. I., TROTTER, S., SINHA, B., RICHMOND, Z. & BURGE, P. S. 2017. Biopsy-proven hypersensitivity pneumonitis caused by a fluorocarbon waterproofing spray. *Occupational medicine,* 67**,** 308-310.

WARREN, C. P. & TSE, K. S. 1974. Extrinsic allergic alveolitis owing to hypersensitivity to chickens-significance of sputum precipitins. *American Review of Respiratory Disease,* 109**,** 672-677.

WEISS, W. & BAUR, X. 1987. Antigens of powdered pearl-oyster shell causing hypersensitivity pneumonitis. *Chest,* 91**,** 146-148.

WELLE, K., BIRSA, M., JELER, E. & ROS SMREKAR, S. 1978. [Our cases of farmer's lung (author's transl)]. *Plucne Bolesti i Tuberkuloza,* 30**,** 159-162.

WELTERMANN, B. M., HODGSON, M., STOREY, E., DEGRAFF, A. C., JR., BRACKER, A., GROSECLOSE, S., COLE, S. R., CARTTER, M. & PHILLIPS, D. 1998. Hypersensitivity pneumonitis: a sentinel event investigation in a wet building. *American Journal of Industrial Medicine,* 34**,** 499-505.

WINCK, J. C., DELGADO, L., MURTA, R., LOPEZ, M. & MARQUES, J. A. 2004. Antigen characterization of major cork moulds in Suberosis (cork worker's pneumonitis) by immunoblotting. *Allergy,* 59**,** 739-745.

WOODARD, E. D., FRIEDLANDER, B., LESHER, R. J., FONT, W., KINSEY, R. & HEARNE, F. T. 1988. Outbreak of hypersensitivity pneumonitis in an industrial setting. *Journal of the American Medical Association,* 259**,** 1965-9.

YAMAMOTO, Y., OSANAI, S., FUJIUCHI, S., AKIBA, Y., HONDA, H., NAKANO, H., OHSAKI, Y. & KIKUCHI, K. 2002. [Saccharomyces-induced hypersensitivity pneumonitis in a dairy farmer: a case report]. *Nihon Kokyuki Gakkai zasshi,* 40**,** 484-8.

YASUI, H., MATSUI, T., YOKOMURA, K., NAKANO, Y., SUDA, T. & CHIDA, K. 2010. [Three cases of hypersensitivity pneumonitis in citrus farmers]. *Nihon Kokyuki Gakkai zasshi,* 48**,** 172-7.

YOSHIDA, K., ANDO, M., ITO, K., SAKATA, T., ARIMA, K., ARAKI, S. & UCHIDA, K. 1990. Hypersensitivity pneumonitis of a mushroom worker due to Aspergillus glaucus. *Archives of Environmental Health,* 45**,** 245-247.

YOSHIDA, K., SUGA, M., NISHIURA, Y., ARIMA, K., YONEDA, R., TAMURA, M. & ANDO, M. 1995. Occupational hypersensitivity pneumonitis in Japan: Data on a nationwide epidemiological study. *Occupational and Environmental Medicine,* 52**,** 570-574.

YOSHIDA, K., SUGA, M., YAMASAKI, H., NAKAMURA, K., SATO, T., KAKISHIMA, M., DOSMAN, J. A. & ANDO, M. 1996. Hypersensitivity pneumonitis induced by a smut fungus Ustilago esculenta. *Thorax,* 51**,** 650-7.

YOSHIDA, K., UEDA, A., YAMASAKI, H., SATO, K., UCHIDA, K. & ANDO, M. 1993. Hypersensitivity pneumonitis resulting from Aspergillus fumigatus in a greenhouse. *Archives of Environmental Health,* 48**,** 260-262.

YOSHIKAWA, S., TSUSHIMA, K., YASUO, M., FUJIMOTO, K., KUBO, K., KUMAGAI, T. & YAMAZAKI, Y. 2007. Hypersensitivity pneumonitis caused by Penicillium citrinum, not Enoki spores. *American Journal of Industrial Medicine,* 50**,** 1010-1017.

YOSHIMURA, N., NODERA, H., OHKOUCHI, M., TSUKIMOTO, K., BEPPU, H., ATARASHI, K., ICHIOKA, M., YOSHIZAWA, Y. & MATSUBARA, O. 1998. [Chronic hypersensitivity pneumonitis due to isocyanate in a patient presenting with acute symptoms 1 month after environmental exposure]. *Nihon Kokyuki Gakkai zasshi,* 36**,** 627-32.

YOSHIZAWA, Y., OHTSUKA, M., NOGUCHI, K., UCHIDA, Y., SUKO, M. & HASEGAWA, S. 1989. Hypersensitivity pneumonitis induced by toluene diisocyanate: sequelae of continuous exposure. *Annals of Internal Medicine,* 110**,** 31-4.

ZACHARISEN, M. & SCHOENWETTER, W. 2005. Fatal hypersensitivity pneumonitis. *Annals of Allergy, Asthma and Immunology,* 95**,** 484-487.

ZACHARISEN, M. C., KADAMBI, A. R., SCHLUETER, D. P., KURUP, V. P., SHACK, J. B., FOX, J. L., ANDERSON, H. A. & FINK, J. N. 1998. The spectrum of respiratory disease associated with exposure to metal working fluids. *Journal of Occupational and Environmental Medicine,* 40**,** 640-647.

ZAMARRON, C., DEL CAMPO, F. & PAREDES, C. 1992. Extrinsic allergic alveolitis due to exposure to esparto dust. *Journal of Internal Medicine,* 232**,** 177-179.

ZEISS, C. R., KANELLAKES, T. M. & BELLONE, J. D. 1980. Immunoglobulin E-mediated asthma and hypersensitivity pneumonitis with precipitating anti-hapten antibodies due to diphenylmethane diisocyanate (MDI) exposure. *Journal of Allergy and Clinical Immunology,* 65**,** 346-352.

ZUBELDIA, J. M., GIL, P., MIRALLES, P., DE BARRIO, M., ARANZABAL, A., HERRERO, T., RUBIO, M., BOUZAS, E. & BAEZA, M. L. 1995. Hypersensitivity pneumonitis caused by soybean antigens. *Journal of Allergy and Clinical Immunology,* 95**,** 622-626.

**Table 1. Occupational sources of hypersensitivity pneumonitis and causative antigens and haptens**

| **Sources** | **Antigen/hapten** | **Disease** | **Reference** | |
| --- | --- | --- | --- | --- |
|  |  |  | **Peer reviewed** | **Non-peer reviewed /conference abstracts** |
| **1. Agricultural work** | | | | |
| Chicory leaves Mould | *Fusarium* sp. | Chicory worker's lung | Colin et al., 2007 |  |
| Citrus farm | *Aspergillus* species *Penicillium* species |  |  | Yasui et al., 2010 |
| Corn | Corn protein |  | Martin-Garcia et al., 2003 |  |
|  | *Aspergillus* species  *Aspergillus flavus* | Farmer’s lung | Patterson et al., 1974, Moreno-Ancillo et al., 2004 |  |
| Cotton work | ? |  |  | Khomenko and Ozerova, 1996 |
| Fruit mould | ? |  |  | Mder et al., 1988 |
| Grain dust | *Arthrobacter globiformis  Alcaligenes faecalis Brevibacterium linens Erwinia herbicola Pantoea agglomerans Staphylococcus epidermidis* |  | Dutkiewicz et al., 1985, Milanowski et al., 1998 |  |
| Grape mould | *Botrytis cinerea* | Winegrower’s lung Späetlase lung | Richerson et al., 1989* |  |
| Hay/straw dust | *Saccharopolyspora rectivirgula Thermoactinomyces vulgaris  Absidia corymbifera Alternaria* *Aspergillus* species *Aureobasidium pullulans Candida* species  *Candida albicans* *Cryptostroma corticae Eurotium amstelodami Mucor* species  *Saccharomyces cerevisiae Wallemia sebi* | Farmer’s lung | Campbell, 1932, Debeljak and Sorli, 1975, Ebner et al., 1981, Frank, 1982, MacLeod and Brown, 1982, Chasse et al., 1986, Rask-Andersen, 1989, Marx Jr et al., 1990, Kokkarinen et al., 1992, Ando et al., 1994, Yoshida et al., 1995, Reboux et al., 2001, Ferri et al., 2003, Thomas et al., 2005, Hanak et al., 2007, Fenclova et al., 2009, Roussel et al., 2010, Koschel et al., 2011, Deschenes et al., 2012, Koschel et al., 2012, Ricco et al., 2019, Tjalvin et al., 2020 | Welle et al., 1978, Barzo et al., 1989, Catenacci et al., 1990, Moura and Pinelo, 2011, Satoh et al., 2011, Brooks et al., 2017 |
| Malt dust | Malt protein |  | Miedinger et al., 2009 |  |
|  | *Aspergillus* species *Aspergillus clavatus Aspergillius fumigatus* | Malt-worker’s lung | Riddle et al., 1968, Channell et al., 1969, Grant et al., 1976, Ellis and Friend, 1981, Thomas, 1995, Fenclova et al., 2009 |  |
| Mushroom production/compost | Thermophilic actinomycetes *Thermoactinomyces vulgaris*  *Aspergillus glaucus Penicillium citrinum Trichosporon cutaneum* | Mushroom-worker’s lung Mushroom grower's disease | Yoshida et al., 1990, Sanderson et al., 1992, Hoy et al., 2007, Yoshikawa et al., 2007 | Brun et al., 1979, Marland et al., 1982, Kishimoto et al., 1993, |
| Mushroom spores | Common mushroom (*Agaricus bisporus*) Eryngi (*Pleurotus Eryngii*) Maitake (*Grifola frondosa*)  Nameko (*Pholiota nameko*) Oyster mushroom (*Pleurotus osteatus*) Shiitake (*Lentinus edodes*) Bunashimeji (*Hypsizigus marmoreus*) Shimeji (*Tricholoma conglobatum*)  *Lyophyllum karst* | Mushroom-worker’s lung | Nakazawa and Tochigi, 1989, Kamm et al., 1991, Ishii et al., 1994, Yoshida et al., 1995, Inage et al., 1996, Murakami et al., 1997, Mori et al., 1998, Akizuki et al., 1999, Tanaka et al., 2000, Suzuki et al., 2001, Tanaka et al., 2001, Saikai et al., 2002, Tanaka et al., 2004, Taniguchi et al., 2004, Tsushima et al., 2005, Tsushima et al., 2006, Hoy et al., 2007, Ampere et al., 2012, Loverdos et al., 2015, Walters et al., 2019 | Konishi et al., 1994, Fujiwara et al., 2000, Tsushima et al., 2000a, Tsushima et al., 2000b, , Miyazaki et al., 2003, Kai et al., 2008, Takaku et al., 2009, , Kalaycioglu and Bicakoglu, 2013, Taha Bekci et al., 2014, |
|  | *Lyophyllum aggregatum*  *Strophariaceae* |  |  | Tsushima et al., 2000a, Tsushima et al., 2000b |
| Onions and potatoes | *Saccharopolyspora rectivirgula* Thermophilic actinomycetes  *Fusarium solani Penicillium* species | Potato riddler's lung | Greene and Bannan, 1985, Merget et al., 2008 |  |
| Plants in greenhouse: vegetables, tomatoes, flowers, roses | *Aspergillus fumigatus Aspergillus glaucus*  *Aspergillus niger Cladosporium herbarum Penicillium* species *Sphaerotheca fuliginea* |  | Yoshida et al., 1993, Yoshida et al., 1995, De Beukelaar et al., 2015, Abreu et al., 2020 | Amano et al., 2009, Hamaguchi et al., 2009 |
| Rockwool used for rose culture | *Aspergillus niger* |  |  | Miyazaki et al., 2004 |
| Silage mould | *Saccharomyces cerevisiae* |  |  | Yamamoto et al., 2002 |
| Smut fungus | *Ustilago esculenta* |  | Yoshida et al., 1996 |  |
| Tobacco mould | ? | Tobacco growers' disease | Khomenko et al., 1989, Nefedov et al., 1991 | Khomenko and Ozerova, 1996 |
| Vineyard fungicide (Bordeaux mixture) | ? | Vineyard-sprayer’s lung | Pimentel and Marques, 1969 |  |
| **2. Processing of plant matter** | | | | |
| Broom grass manufacturing | *Calluna valgaris* |  | Aydemir et al., 2015 |  |
| Coir industry | ? |  |  | Shanmugapriya et al., 2013 |
| Esparto (*Stipa tenacissima*) dust | Esparto grass protein  *Aspergillus* species *Aspergillus fumigatus Mucor* sp. *Penicillium frequentans* | Stipatosis Espartosis | Gamboa et al., 1990, Zamarron et al., 1992, Morell et al., 1995, Hinojosa et al., 1996, Moreno-Ancillo et al., 1997, Cruz et al., 2003, Moreno-Ancillo et al., 2003, Flandes et al., 2004 |  |
| Green tea | Green tea protein |  | Tanaka et al., 2016 | Sano et al., 2004 |
| Non-sterile argan (*Argania spinosa*) powder | ? |  | Paris et al., 2015 |  |
| Peat moss processing | *Monocillium* sp. *Penicillium citreonigrum* |  | Cormier et al., 1998 |  |
| Seaweed | *Sphaerotheca fuliginea* |  | Henderson et al., 1984 |  |
| Soybean hulls | Soybean protein |  | Zubeldia et al., 1995 |  |
| Spinach powder | Spinach protein |  | Schreiber et al., 1998 |  |
| Straw mat | Moulds |  | Yoshida et al., 1995 |  |
| Sugar cane residue | Thermophilic actinomycetes *Thermoactinomyces vulgaris*  *Thermoactinomyces sacchari* | Bagassosis | Torres Jimenez et al., 1979, Yoshida et al., 1995, Walters et al., 2019 |  |
| Thyme dust | *Pantoea agglomerans* |  | Mackiewicz et al., 1999 |  |
| Tiger nut | Tiger nut protein |  | Barranco et al., 1999 |  |
| Tobacco mould | *Aspergillus* sp. | Tobacco-worker’s lung | Huuskonen et al., 1984, Lander et al., 1988 |  |
| **3. Wood and woodwork** | | | | |
| Bark mulch | *Cryptostroma corticale Phanerochaete chrysosporium* |  | Shepherd et al., 1989, Lanspa and Hatton, 2014 |  |
| Cork dust | Suberin | Suberosis | Morell et al., 2003 |  |
|  | *Aspergillus fumigatus Mucor* species  *Penicillium frequentans (Penicillium glabrum)* | Suberosis | Moral et al., 1994, Morell et al., 2003, Winck et al., 2004, Villar et al., 2009 | Neddam et al., 1998 |
| Fuel chip dust | *Aspergillus fumigatus Mucor* species *Paecilomyces* species |  | Muller-Wening et al., 1999 |  |
| Maple bark | *Cryptostroma corticale* | Maple bark–stripper’s lung | Richerson et al., 1989* |  |
| Sequoia (Redwood) dust | *Aureobasidium pullulans Graphium* | Sequoiosis | Cohen et al., 1967 |  |
| Wood dust or wood pulps | *Thermoactinomyces vulgaris  Alternaria Aspergillus* species *Paecilomyces Penicillium* sp*. Penicillium chrysogenum*  *Rhizopus icrospores Trichoderma koningii* | Woodworker’s lung Woodman’s disease Wood trimmer’s disease Wood-pulp workers’ disease | Schlueter et al., 1972, Terho et al., 1980, Dykewicz et al., 1988, Halpin et al., 1994, Rask-Andersen et al., 1994, Veillette et al., 2006, Faerden et al., 2014 | Haahtela et al., 1979 |
| Wood | Cabreuva (*Myrocarpus fastigiatus*) protein Pine protein Ramin (*Gonystylus bancanus*) protein |  | Howie et al., 1976, Baur et al., 2000 |  |
| **4. Animal-related work**s | | | | |
| Animal feed | Phytase |  | Van Heemst et al., 2009 |  |
| Animal fur | Animal fur protein: fox, karakul | Furrier’s lung | Pimentel, 1970 |  |
|  | *Trichothecium roseum* | Furrier’s lung |  | Helbecque et al., 1991 |
| Avian droppings, blood, or feathers | Avian protein: budgerigars, canary, chicken, cockatoos, hill mynahs, pigeon, pheasant, wood bird, owls | Bird-breeder’s lung Bird-fancier’s lung Pheasant rearer’s lung Pigeon-breeder’s lung Poultry worker’s lung | Fink et al., 1968, Ávila, 1971, Warren and Tse, 1974, Bergmann Ch, 1979, Reheda, 1994, Choy et al., 1995, Yoshida et al., 1995, Inase et al., 2004, Partridge et al., 2004, Bocchia et al., 2005, Zacharisen and Schoenwetter, 2005, Mehta et al., 2008, Holden et al., 2017, Walters et al., 2019 | Khomenko and Ozerova, 1996, Suga and Sukoh, 2002, Ishiguro et al., 2010, Barrett and Hayes, 2012, Moldovan et al., 2019 |
| Bovine heads | Bovine’s pituitary glands protein |  |  | Gala Ortiz et al., 1999 |
| Carmine (red dye) | *Coccus cactus* scale protein |  | Dietemann-Molard et al., 1991 |  |
| Clam | ? |  | Gumundsson et al., 2002 |  |
| Feather duvet manufacturing | Pigeon protein |  |  | Fujie et al., 2004 |
| Fish meal dust | Fish protein | Fishmeal-worker’s lung | Ávila, 1971 |  |
| Gerbil | Gerbil protein | Gerbil-keeper’s lung | Riario Sforza and Marinou, 2017* |  |
| Pig serum or skin | Pig protein | Pig breeder's lung |  | Schlegel et al., 1990 |
| Rat serum or urine | Rat protein | Rodent-handler’s lung | Carroll et al., 1975 |  |
| Salmon | Salmon protein |  | Tjalvin et al., 2018 | Kirkeleit et al., 2017 |
| Silkworm larvae | Silkworm larvae protein | Sericulturist’s lung | Nakazawa and Umegae, 1990, Yoshida et al., 1995 |  |
| Shell dust | Shell dust protein |  | Yoshida et al., 1995, |  |
|  | Mollusc shell: pearl-oyster, sea-snail | Oyster shell lung Mollusc shell HP | Weiss and Baur, 1987, Orriols et al., 1990, Jager et al., 1991, Mitani et al., 1995, Orriols et al., 1997 |  |
| **5. Foodstuff** |  |  |  |  |
| Baker’s yeast | *Saccharomyces cerevisiae* |  | Gernhold and Sennekamp, 2010 |  |
| Cheese | *Penicillium casei Pencillium roqueforti* | Cheese-washer’s lung | Schlueter, 1973, Campbell et al., 1983, Galland et al., 1991 | Brun et al., 1979, Catenacci et al., 1990 |
| Dry sausage dust | *Aspergillus fumigatus*  *Penicilluim* species *Penicillium camembertii*  *Penicillium frequentans* | Chacinero’s lung Salami worker's lung | Reynaud et al., 1992, Marchisio et al., 1999, Rivero et al., 1999, Guillot et al., 2008, Morell et al., 2011 |  |
| Flour | Wheat weevil protein | Miller’s lung | Lunn and Hughes, 1967 |  |
|  | Flour mite (*Acarus siro*)  *Aspergillus fumigatus* |  | Gerfaud-Valentin et al., 2014 |  |
| Buckwheat flour | ? |  | Yoshida et al., 1995 |  |
| Paprika | *Mucor stolonifer* | Paprika-splitter’s lung | Riario Sforza and Marinou, 2017* |  |
| Spice dust | ? |  | Shivanthan and Wijesiriwardena, 2011 |  |
| **6. Food processing** |  |  |  |  |
| Imitation cheese manufacturing | ? | Cheese Whiz lung |  | Bringgold and Halliday, 2020 |
| Koji brewery | *Aspergillus oryzae* |  | Ishiguro et al., 2018 |  |
| Konnyaku manufacturing | Konjak flour *Hijikia fusiforme* | Konnyaku manufacturer's lung | Tajima et al., 2003 |  |
| Soy sauce brewery | *Aspergillus oryzae* |  | Tsuchiya et al., 1993, Yoshida et al., 1995 |  |
| Coffee bean dust | Coffee protein | Coffee-worker’s lung | van Toorn, 1970, Boyadzieva et al., 2014 |  |
| **7. Metals and metal products** | | | | |
| Dust in a foundry’s ceiling light | *Thermoactinomyces vulgaris* |  | Valbuena et al., 1993 |  |
| Metal-working fluid | Acinetobacter *Mycobacterium* species  *Mycobacterium avium Mycobacterium chelonae Mycobacterium immunogenum Ochrobactrum Pseudomonas oleovorans* | Machine operator's lung Machine-worker’s lung Metal worker's lung | Zacharisen et al., 1998, Fox et al., 1999, Shelton et al., 1999, Hodgson et al., 2001, Bracker et al., 2003, O'Brien, 2003, Beckett et al., 2005, Dawkins et al., 2006, Robertson et al., 2007, Gupta and Rosenman, 2006, Tillie-Leblond et al., 2011, James et al., 2018, Bellanger et al., 2019 | Centers for Disease Control and Prevention, 1996, Centers for Disease Control and Prevention, 2002, Omura et al., 2012, James et al., 2015 |
| Water-based metal-working fluid | *Bacillus pumilus Pseudomonas fluorescens Pseudomonas pseudoalcaligene Rhodococcus sp Staphylococcus capitas Aspergillus* species *Aspergillus niger* |  | Bernstein et al., 1995, Freeman et al., 1998, Moniodis et al., 2015  Zacharisen and Schoenwetter, 2005 |  |
| Metal smelting | Zinc |  | Ameille et al., 1992 |  |
| Mould preparation | Diphenylmethane diisocyanate |  | Malo and Zeiss, 1982 |  |
| Steel plant | Diphenylmethane diisocyanate |  | Walker et al., 1989 |  |
| Welding | Zinc fumes |  |  | Miyazaki et al., 2006 |
| **8. Polymers, plastics, and fibres** | | | | |
| Adhesives | Diphenylmethane diisocyanate  Toluene diisocyanate |  | Baur, 1995 |  |
| Cold cure adhesive | Triphenylmethane Triisocyanate |  | Buick and Todd, 1997 |  |
| Bathtub refinishing process | Toluene diisocyanate | Bathtub refinisher’s lung | Fink and Schlueter, 1978 |  |
| Elastic brick production | Diphenylmethane diisocyanate |  | Fenclova et al., 2009 |  |
| Foam production | Isocyanates:  tris(4-isocyanatophenyl)-thiophosphate  Diphenylmethane diisocyanate | Chemical-worker’s lung Plastic worker’s lung Epoxy-worker’s lung | Baur, 1995 |  |
| Glues | Isocyanates |  | Bartizalova, 2012 | Unsal et al., 2014 |
| Injection moulding | Diphenylmethane diisocyanate |  | Baur, 1995 |  |
| Paints/Spray paints | Hexamethylene diisocyanate  Diphenylmethane diisocyanate  Toluene diisocyanate | Paint-refinisher’s lung Spray painter's lung | Baur, 1995, Yoshizawa et al., 1989, Usui et al., 1992, Forst and Abraham, 1993, Oh et al., 2008, Schreiber et al., 2008 | Nozawa et al., 1989, Akimoto et al., 1992, Bando et al., 1993, Nakamura et al., 1995, Hashizume et al., 2001, Matsushima et al., 2003, Cloete, 2014 |
| Polyurethane paints | Hexamethylene diisocyanate Diphenylmethane diisocyanate  Toluene diisocyanate |  | Yoshida et al., 1995, | Idezuka et al., 1991, Saak and Stresemann, 1994, Yoshimura et al., 1998 |
| Polyester powder paints | Phthalic anhydride  Triglycidyl isocyanurate Trimellitic anhydride |  | Piirila et al., 1997, Quirce et al., 2004 |  |
| Polyethylene terephthalate (PET) production | Dimethyl terephthalate  Terephthalic acid |  | Sartorelli et al, 2020 |  |
| Polyurethane related industry | Diisocyanates: Diphenylmethane diisocyanate Isophane diisocyanate Naphthylene-1,5-diisocyanate |  | Zeiss et al., 1980, Bergh, 1982, Baur et al., 1984, Yoshida et al., 1995, Merget et al., 2002, Germanaud et al., 2003, Lhoumeau et al., 2012 | Hara et al., 2009 |
| Surface coating |  |  |  |  |
| Epoxy resin system chemical | ? | Epoxy resin workers | Hines et al., 2015 |  |
| HDI-based hardener | Hexamethylene diisocyanate (HDI) |  | Bieler et al., 2011 |  |
| Synthetic fibres | ? |  | Pimentel et al., 1975 |  |
| **9. Other manufacturing** |  |  |  |  |
| Aerospace industry | Acid anhydrides |  | Quirce et al., 2016* |  |
| Automotive industry | Naphthylene-1,5-diisocyanate |  | Baur et al., 2001 |  |
| Ceramic industry | Zirconium |  | Liippo et al., 1993 |  |
| Detergent industry | *Bacillus subtilis* enzymes (subtilisin) | Detergent-worker’s lung | Tripathi and Grammer, 2001, Schulte et al., 2008, Benzarti Mezni et al., 2010 |  |
| Nuclear technology plant | Zirconium (welding fumes) |  |  | Schneider et al., 1994, |
| Pharmaceutical industry | Penicillin |  | De Hoyos et al., 1993 |  |
| Shoe-making process | ? | Shoemaker’s EAA |  | Poliakova, 1992 |
| Yacht manufacturing | Dimethyl phthalate  Styrene | Yacht-maker's lung | Volkman et al., 2006 |  |
| **10. Miscellaneous chemicals and synthetic substances** | | | | |
| Colophony flux-cored solder | Colophony fumes |  | Maria et al., 1992 |  |
| Coolants |  |  |  |  |
| Diode-laser coolants | 1,1,1,2-tetrafluoroethane (HFC134a) |  | Ishiguro et al., 2007 |  |
| Water-based coolants | *Aspergillus* species |  | Zacharisen and Schoenwetter, 2005 |  |
| Deterzyme | Protolytic enzymes amylases of *Aspergillus oryzae* |  | Gueland et al., 1993 |  |
| **11. Work with aerosolized water** |  |  |  |  |
| Air conditioners, cooling water | *Cytophaga endotoxin Thermoactinomyces vulgaris  Aspergillus* species *Aspergillus fumigatus Aureobasidium pullulans Cladosporium cladosporiodes Penicillium Rhizopus* species Slime mould | Office worker’s lung Air conditioner’s lung | Friend et al., 1977, Arnow et al., 1978, Storms, 1978, Bernstein et al., 1983, Reed et al., 1983, Hodgson et al., 1987, Woodard et al., 1988, O'Connell et al., 1995, Yoshida et al., 1995, Nordness et al., 2003, Chiba et al., 2009, Magnavita, 2013, D'Souza and Donato, 2017, Walters et al., 2019 |  |
| Humidifiers | *Aspergillus fumigatus Candida* species *Cephalosporium acremonium Penicillium* species *Sporothrix schenckii*  Amoebas | Humidifier lung | Van Assendelft et al., 1979, Ganier et al., 1980, Solley and Hyatt, 1980, Gerber et al., 1981, Robertson et al., 1987, Yoshida et al., 1995, Pal et al., 1997, Walters et al., 2019 | Sakurai et al., 2001 |
| Spas and hot tubs | *Mycobacterium avium* complex | Hot tub lung | Moraga-McHaley et al., 2013, Rudrappa and Kokatnur, 2017 |  |
| Vaporzone | *Pseudomonas* sp. |  | Soumagne et al., 2016 |  |
| **12. Service work** |  |  |  |  |
| Archiving | Moulds |  | Walters et al., 2019 |  |
| Banknotes | ? | Hard cash HP | Kupeli et al., 2010 |  |
| Damp building (office, school) | *Aspergillus fumigatus* | Office-related HP | Thorn et al., 1996, Weltermann et al., 1998 |  |
| Degreasing agents (computer repairing work) | Trichloroethylene |  | Kim et al., 2014 |  |
| Dental work | Acrylate compounds Methyl methacrylate |  | Scherpereel et al., 2004 |  |
| Dry cleaning process | Tetrachloroethylene |  | Tanios et al., 2004 |  |
| Fluorocarbon waterproofing aerosol spray (used in furniture retail) | Fluorocarbon |  | Walters et al., 2017 |  |
| Frankincense (sambrani) fumes | ? |  |  | Shanmugapriya et al., 2013 |
| Hair bleach | Persulphate salts |  |  | Svanes et al., 2014 |
| Hairdressing work | *Trichosporon cutaneum* |  | Kawane and Soejima, 1987 |  |
| Human nail dust | ? |  |  | Sharma et al., 2013 |
| Human skin and nails | *Aspergillus fumigatus*  *Candida albicans Candida glabrata Penicillium brevicompactum* | Chiropody alveolitis | Lingenfelser and Sennekamp, 2010 |  |
| Insecticides (from freight) | Zetacypermethrin | Insecticide lung |  | Nahata and Essen, 2013 |
| Laundry work | Moulds |  | Yoshida et al., 1995 |  |
| Medical office | Indoor mould |  | Mayer et al., 1990 |  |
| Pet groomer spray | Pyrethrin | Pet Groomer's Lung | Pu et al., 2017 |  |
| Sodium diazobenzenesulphate (Pauli's reagent) in medical laboratory | Sodium diazobenzenesulphate | Pauli’s reagent lung | Evans and Seaton, 1979 |  |
| **13. Waste and sewage** |  |  |  |  |
| Compost | Thermophilic actinomycetes *Thermoactinomyces vulgaris Streptomyces albus  Aspergilllus* sp. | Composter’s lung | Kagen et al., 1981, Vincken and Roels, 1984, Bunger et al., 2007, Lal et al., 2018 |  |
| Household waste or recycling | *Actinomycetes*  *Aspergillus fumigatus* |  | Hagemeyer et al., 2013, Walters et al., 2019 |  |
| Organic waste | *Aspergillus fumigatus Penicillium chrysogenum* |  | Preisser and Harth, 2017 |  |
| **14. Wind instruments** |  |  |  |  |
| Bagpipes | ? Microorganisms found in bagpipe: *Exophiala dermatitidis*  *Fusarium oxysporum*  *Paecilomyces variotii Penicillium* species *Rhodotorula mucilaginosa Trichosporon mucoides* Pink yeast |  | King et al., 2017 |  |
| Saxophone | *Phoma* sp. *Ulocladium botrytis* | Sax lung | Metzger et al., 2010 |  |
| Trombone | ? Microorganisms found in trombone: *Escherichia coli*  *Mycobacterium chelonae-abscessus Stenotrophomonas maltophilia*  *Fusarium* sp. | Trombone player's lung | Metersky et al., 2010 |  |

* review article

? Not identified or reported
